# Unconventional Electron-Deficient Multicenter Bonds in AIO_3_ Perovskites

**DOI:** 10.1021/acs.chemmater.5c00877

**Published:** 2025-05-30

**Authors:** Hussien H. Osman, José Luis Rodrigo-Ramón, Shafi Ullah, Enrico Bandiello, Daniel Errandonea, Óscar Gomis, Tania García-Sánchez, Pablo Botella, Robert Oliva, Plácida Rodríguez-Hernández, Alfonso Muñoz, Catalin Popescu, Frederico G. Alabarse, Francisco Javier Manjón

**Affiliations:** † Instituto de Diseño para la Fabricación y Producción Automatizada, MALTA Consolider Team, 16774 Universitat Politècnica de València, 46022 València, Spain; ‡ Instituto de Ciencia de los Materiales de la Universitat de València, MALTA Consolider Team, Universitat de València, 46100 Valencia, Spain; § Chemistry Department, Faculty of Science, Helwan University, Cairo 11795, Egypt; ∥ Centro de Tecnologías Físicas, MALTA Consolider Team, Universitat Politècnica de València, 46022 València, Spain; ⊥ Geosciences Barcelona (GEO3BCN), MALTA Consolider Team, CSIC, Lluís Solé i Sabarís s/n, 08028 Barcelona, Catalonia Spain; # Departamento de Física, MALTA Consolider Team, 16749 Universidad de La Laguna, 38205 La Laguna, Tenerife , Spain; ∇ ALBA-CELLS, MALTA Consolider Team, 08290 Cerdanyola del Valles (Barcelona), Catalonia , Spain; ○ Elettra Sincrotrone Trieste, S.S. 14 - Km 163,5 in AREA Science Park, 34149 Basovizza, Trieste , Italy

## Abstract

*ABX*_3_ *and BX*_3_ perovskites and their distorted variants are solidstate systems with exceptional properties, which allow them to be used in a plethora of potential technological applications. This notwithstanding, the nature of the chemical *B*–*X* bonding, which forms the framework where the *A* atoms can be inserted, is still under debate. Through a joint experimental and theoretical study of *A*IO_3_ (*A* = K, Rb, Cs, Tl, NH_4_) compounds and in particular in cesium iodate (CsIO_3_) under compression, we show how the IO_3_^–^ polyanions, present in these compounds at room pressure, undergo a gradual pressure-induced polymerization (PIP) process in three dimensions (3D). This results in a pressure-induced symmetrization of the crystalline structure that leads to a tetragonal perovskite structure, with IO_5+1_ units, in CsIO_3_ and eventually to a cubic perovskite, with IO_6_ units, in other *A*IO_3_ compounds. We demonstrate that the PIP process induces a change in the chemical bonding from the resonant delocalized I–O bonds in IO_3_^–^ polyanions toward the unconventional I–O electron-deficient multicenter bonds (EDMBs) in *A*IO_3_ cubic perovskites. The process of EDMB formation in the cubic perovskites agrees with the recently proposed unified theory of multicenter bonding and contradicts previous assumptions that considered these bonds to be impossible in valence electron-rich elements, such as chalcogens and halogens. Interestingly, our results suggest that (i) the formation of the cubic and slightly distorted *ABX*_3_ and *BX*_3_ perovskites, with *A*, *B*, and *X* being main-group elements, at high pressure is driven by the formation of 3D EDMBs due to the PIP process of the *BX*_3_ units (monomers) leading to the formation of regular *BX*_6_ units; and (ii) unconventional EDMBs could be already present at room conditions in the cubic or slightly distorted *ABX*_3_ and *BX*_3_ perovskites, with *A*, *B*, and *X* being main-group elements. The presence of unconventional EDMBs could explain the extraordinary properties of these perovskites.

## Graphical abstract

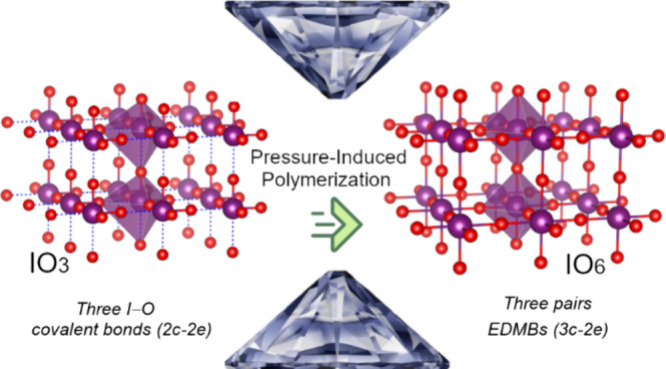

## 1 Introduction

The perovskite (PV) structure can be found in *ABX*_3_ and *BX*_3_ compounds and is perhaps the single most versatile ceramic host since appropriate changes in composition can transform the most significant electro-ceramic dielectric phase in the industry, BaTiO_3_, into metallic conductors, superconductors, catalysts, sensors, lasers, magnetoresistive/multiferroic/thermoelectric materials, and even high-performance photovoltaic materials, such as lead halide PVs.^1–4^ The rich diversity of chemical compositions and properties of PVs results in “high-tech” applications, which have made PVs to be dubbed *inorganic chameleons*.

PVs are also very important materials from a fundamental perspective, and still, they represent an exciting challenge in terms of chemical bonding. If we consider *ABX*_3_ PVs with *A*, *B*, and *X* being main-group elements, e.g. CsPbI_3_, it is clear that there is mainly an ionic *A*–*X* bonding. However, it must be noticed that a certain covalent character has been observed in the *A*–*X* bonding of certain PVs when *A* is a group-15 element (e.g., Pb in PbTiO_3_).^5^ On the other hand, the nature of the chemical *B*–*X* bonding, which forms the ReO_3_-type octahedral framework in which *A* atoms are inserted,^6–8^ is a mystery to be unraveled in both *ABX*_3_ and *BX*_3_ PVs of main-group elements.

Claims for a mixture of classical ionic, covalent, and even metallic bonding have been proposed to be present in *B*–*X* bonds to explain the extraordinary properties of *ABX*_3_ and *BX*_3_ PVs.^9,10^ Recently, the debate regarding the chemical *B*–*X* bonding in PVs has been renewed since Wuttig and co-workers have suggested that lead halide PVs, like CsPbI_3_, feature an unconventional new type of two-center-one-electron (2c–1e) bonding different from the classical (covalent, ionic, and metallic) ones, dubbed as metavalent bonding.^11^ Noteworthy, this new brand bonding is the same that is supposed to explain the extraordinary properties of phase change materials.^12^ In this context, Wuttig and co-workers have suggested that oxide perovskites, like BaTiO_3_, do not have this kind of unconventional bonding (suggesting that the Ti–O bond is of covalent type).^11^

Contrarily, other researchers consider that the metavalent bonding is simply the century-old electron-rich multicenter bonding (ERMB), also known as the hypervalent bonding or the three-center-four-electron (3c–4e) bonding,^13^ and propose that the extraordinary properties of phase change materials is explained in terms of the ERMBs.

To complete the picture, Manjón and co-workers, who have proposed a unified theory of multicenter bonding,^14,15^ have suggested that the unconventional bonding of phase change materials and lead halide PVs is neither the metavalent bonding nor the ERMB, but just the also century-old electron-deficient multicenter bonding (EDMB) occurring in solids with electron-rich elements.^14,15^ These EDMBs are just a linear or quasi-linear concatenation of three-center-two-electron (3c–2e) bonds in which a single electron is present between two atoms; i.e., a 2c–1e bond, as shown by Wuttig and co-workers.

The proposal of Manjón and co-workers that the unconventional EDMB is present in phase change materials and lead halide PVs is based on different bonding descriptors and the behavior of those descriptors with increasing electron density.^14,15^ One of the bonding descriptors is the combination of the number of electrons shared (ES) and the normalized number of electrons transferred (ET) between two atoms, as obtained from the Quantum Theory of Atoms in Molecules.^16^ In the unified theory of multicenter bonding, Manjón and co-workers have shown that the ES and ET values are different for the two multicenter bondings (ERMBs and EDMBs), so they can be located in different regions of the ES vs ET map.^14,15^ Several arguments of Manjón and co-workers demonstrate that metavalent bondings are the old EDMBs and not the old ERMBs: (i) the region in the ES vs ET map for the EDMB is the same already attributed to the new metavalent bonding and completely different to that for the ERMB; and (ii) the two multicenter bonds can be fully distinguished because they have a different formation mechanism. Despite both multicenter bonds share a common origin, their formation proceeds through different mechanisms.

The unified theory of multicenter bonding also suggests that the increase in electron density, either by applying high-pressure (HP) conditions, reduction (i.e., injection of electrons in a system), or substitution of elements by their heavier analogs, helps to form unconventional multicenter bonds. Both ERMBs and EDMBs can be formed by a polymerization process, in which multicenter bonds are formed from original primary and secondary bonds. The primary bonds are usually iono-covalent bonds, although we have recently shown that pressure-induced EDMBs can be formed in AX_3_ compounds when primary bonds are ERMBs.^17^ On the other hand, secondary bonds are usually noncovalent interactions related to the presence of stereochemically active lone electron pairs (LEPs), typically leading to asymmetric structures at room pressure (RP).^18–23^ Importantly, ERMBs and EDMBs have a different formation mechanism because the formation of the ERMB involves the transformation of the nonbonding electrons present in the LEP of secondary bonds into bonding electrons in the ERMB, so the charge for the new bonds is provided by a LEP. On the contrary, the formation of the EDMB is mainly due to the charge transfer from the primary iono-covalent bond to the secondary noncovalent interaction. In other words, the charge for the new EDMB is provided by the strong bond, and, in general, the LEP becomes stereochemically inactive during the formation of the EDMB, so the nonbonding electrons of the LEP remain as nonbonding electrons when the EDMB is formed.^14,15^

The present work aims to show that EDMBs do occur in main-group PVs, in particular in some oxide PVs, such as those formed by *A*IO_3_ (*A* = K, Rb, Cs, Tl, NH_4_) compounds at HP. For this purpose, we have selected CsIO_3_, a compound with a strongly distorted PV structure, and conducted a joint experimental and theoretical study of this compound under compression which is complemented with ab initio calculations in related *A*IO_3_ compounds. The choice of CsIO_3_ to conduct this study is justified because it is a member of the *A*IO_3_ family and this family was theoretically predicted to gradually become closer to the cubic PV structure under compression.^24^ In particular, RbIO_3_ was shown to progressively transform from the distorted rhombohedral PV structure (space group (SG) *R*3*m*, No. 160) to the perfect cubic PV structure (SG *Pm* $\overset{-}{3}$ *m*, No. 221) at moderate pressures (above 35 GPa).

The compounds of the *A*IO_3_ family have been profusely studied because they show ferroelectric and nonlinear optical properties,^25–30^ related to their strongly distorted PV structures.^28,31–36^ The strong distortion of the IO_6_ octahedra in *A*IO_3_ compounds at RP results in molecular compounds with IO_3_ units and a much smaller symmetry than the cubic PV structure. KIO_3_ crystallizes in a triclinic noncentrosymmetric (space group (SG) *P*1, No. 1) structure^31–33^ and NH_4_(IO_3_) does it in an orthorhombic (SG *Pna*2_1_, No. 33) structure.^34^ Less distorted phases are found in *A*IO_3_ (*A* = Rb, Cs, Tl) compounds, which crystallize in the rhombohedral (SG *R*3*m*, No. 160) structure at RP.^28,35,36^ Noteworthy, the rhombohedral *R*3*m* phase is also a high-temperature phase of KIO_3_ at RP.^29^

In the cubic PV structure, all the *B–X* bonds of the *BX*_6_ units have the same bond length; however, in the strongly distorted PV structure of CsIO_3_, there are two I*–*O bond lengths (see Figure 1a): one short I*–*O bond length, which corresponds to three delocalized resonant (covalent-like) bonds that define a trigonal pyramidal IO_3_ unit, and one long I*–*O bond length, which corresponds to three interactions that can be considered as secondary noncovalent bonds in a strongly distorted IO_6_ octahedron (indeed an IO_3+3_ unit).

**Figure 1:**
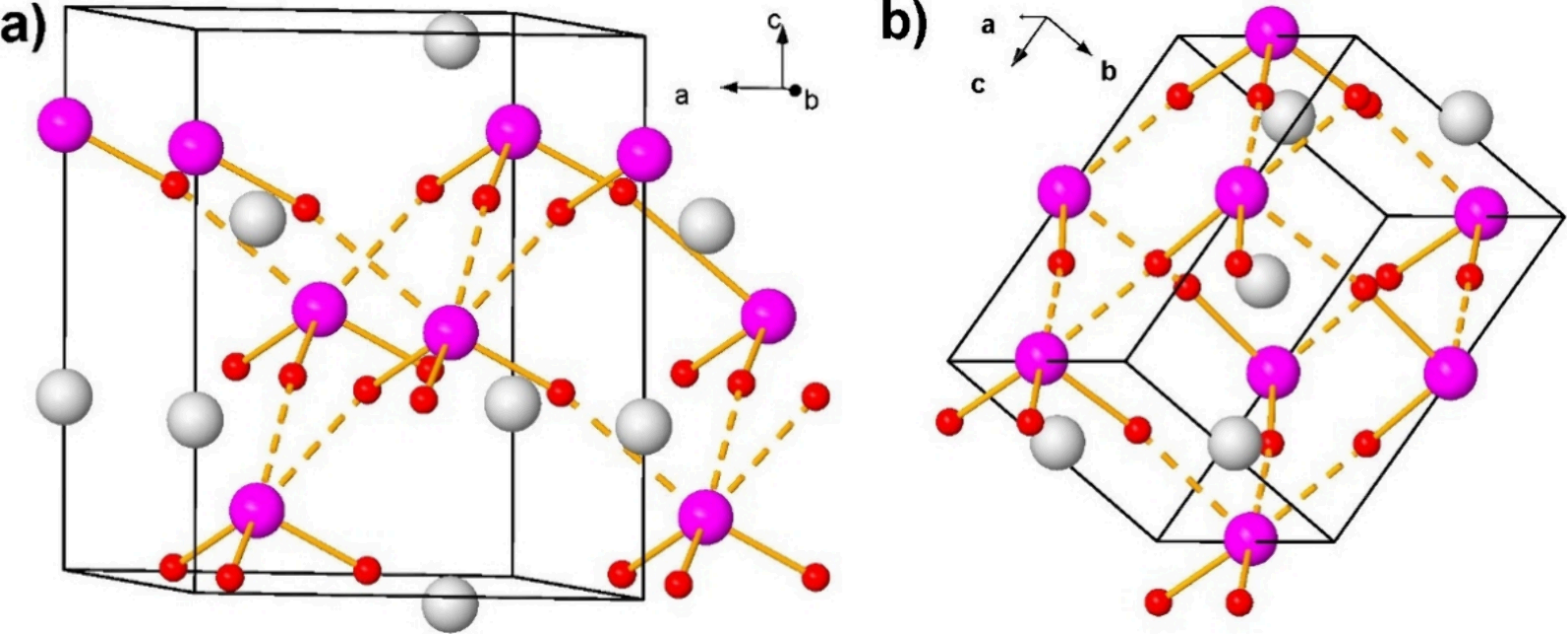
Crystal structure of CsIO_3_ in (a) the *R*3*m* phase at 0 GPa and (b) the *Pmn*2_1_ phase at 20 GPa. Cs, I, and O atoms are plotted in gray, pink, and red, respectively. Short and long bonds are noted as solid and dashed orange lines. The IO_3_ trigonal pyramids can be observed. The perspective of both structures has been selected to highlight their similitudes.

To our knowledge, the *A*IO_3_ family of distorted PVs has not been studied in deep under compression, with only one experimental study on KIO_3_ ^37^ and one theoretical study on RbIO_3_ ^24^ being available. In both works, a pressure-induced symmetrization (PIS) of the distorted PV structure was found to be driven by the regularization of the distorted IO_6_ units. In KIO_3_, two pressure-induced phase transitions were observed at 7 and 14 GPa under hydrostatic conditions. Under nonhydrostatic conditions, these transitions occurred at significantly lower pressures (4 and 10 GPa).^37^ The first HP phase of KIO_3_ was identified as a rhombohedral structure (SG *R*3, No. 146). On the other hand, RbIO_3_ was theoretically predicted to transform from the rhombohedral to the cubic PV structure (SG *Pm* $\overset{-}{3}$ *m*, No. 221) beyond 35 GPa.^24^ Notably, a PIS of the distorted IO_6_ units has also been observed in several metal iodates,^38–43^ as recently reviewed.^44^

In relation to the PIS of the distorted IO_6_ units in iodates, several recent studies on metal iodates have evidenced a pressure-induced change in chemical I–O bonding with the increase in I coordination (hypercoordination).^38–41^ Specifically, the delocalized resonant (covalent-like) I–O bonds in IO_3_ units at RP have been suggested to transform into EDMBs at HP.^41^ We have to note in passing that some of us previously referred to EDMBs as metavalent bonds in earlier studies on metal iodates under compression;^38–40,44^ this was due to the claimed electron-deficient character of the metavalent bond just before it was clarified by Manjón and co-workers that the metavalent bonds are 2c–1e EDMBs that result from the linear (or quasi-linear) concatenation of 3c–2e bonds in solids with electron-rich elements.^14,15,45^

In this work, we present a joint HP experimental and theoretical study of the distorted PV structure of CsIO_3_ to study the change in chemical I*–*O bonding during the PIS of the crystalline structure of CsIO_3_. For this study, we have combined experimental X-ray diffraction (XRD) and Raman scattering (RS) measurements, ab initio calculations, and a detailed electron topology analysis in several members of the *A*IO_3_ family. Our results reveal that CsIO_3_ undergoes several pressure-induced phase transitions driven by the pressure-induced polymerization (PIP) process of the IO_3_ units, which are the monomers that become linked upon pressure increase. As a consequence of this process, the original rhombohedral phase (SG *R*3*m*, No. 160) undergoes a phase transition to an orthorhombic structure (SG *Pmn*2_1_, No. 31). In addition, a second HP phase with a tetragonal structure (SG *P*4*/nmm*, No. 129) is theoretically predicted beyond 45 GPa. We demonstrate that the PIP process leads to a progressive PIS of the structure and a change in the chemical I–O bonding with the increase in I coordination. In particular, we show that two of the three short delocalized resonant I–O bonds of IO_3_ units at the *R*3*m* phase at RP progressively transform (together with two secondary noncovalent bonds) into two EDMBs as pressure increases and the tetragonal PV phase (with IO_5+1_ units) is formed in CsIO_3_. Our findings align with the recently developed theory of multicenter bonding^14,15^ and contradict previous assumptions that considered EDMBs to be impossible in valence electron-rich elements, such as chalcogens and halogens. Interestingly, our findings suggest that the cubic PV structure in *ABX*_3_ and *BX*_3_ compounds with main-group elements, e.g., in *A*IO_3_ compounds at HP, is likely characterized by the presence of EDMBs among the regular *BX*_6_ units that result from the polymerization of *BX*_3_ units.

## 2 Experimental Details

### 2.1 Synthesis and Characterization at Room Pressure

Cesium iodate (CsIO_3_) powders were synthesized from the reaction of cesium carbonate (Cs_2_CO_3_) and iodic acid (HIO_3_). All starting materials, including Cs_2_CO_3_ (99.9%) and HIO_3_ (99.5%), were purchased from Sigma-Aldrich and used without further purification. Cs_2_CO_3_, with a molar mass of 325.82 g/mol, was weighed to achieve a 0.004 M concentration, corresponding to 1.31 g. HIO_3_, with a molar mass of 175.91 g/mol, was similarly prepared at a 0.004 M concentration, equating to 0.704 g. Each substance was dissolved separately in 15 mL of distilled water and stirred for 10 min. The solutions were then combined to produce a total volume of 30 mL and mixed for an additional 10 min. The resulting mixture was transferred to a 40 mL Teflon-lined stainless-steel autoclave and heated in an electric oven at 220 °C for 48 h. The obtained powder was washed several times with distilled water to remove impurities and then dried in an electric oven at 100 °C for 24 h.

The purity of the samples was determined via powder XRD analysis performed using a Rigaku Ultima IV diffractometer in the Bragg–Brentano configuration with CuKα radiation (λ = 1.54060 Å) at room temperature. Data were collected over a 2θ range of 10–70° with a step size of 0.02° and a fixed counting time of 1 s per step. A single phase corresponding to the rhombohedral phase of CsIO_3_ was found, thus confirming the purity of the synthesized samples.

### 2.2 Characterization at High Pressure

HP experiments on powders of CsIO_3_ were conducted utilizing a membrane-style diamond-anvil cell having diamonds with anvils measuring 350 μm in diameter. Stainless-steel gaskets preindented to a thickness of 40 μm, with a 120-μm diameter hole at the center, were employed. As a pressure-transmitting medium, we used a mixture of methanol, ethanol, and water with a ratio 16:3:1, which ensures quasi-hydrostatic conditions up to ca. 10 GPa.^46^ In all experiments, the diamond-anvil cell loading was performed avoiding sample bridging between the diamonds.^47^

#### 2.2.1 HP-XRD Experiments

We performed two HP-XRD experiments in CsIO_3_. One was carried out at the Xpress beamline of Elettra Synchrotron Radiation Facility up to 34.9(1) GPa and the other was conducted up to 40.6(1) GPa at the BL04-MSPD beamline of ALBA synchrotron.^48^ At Elettra (ALBA) we used a monochromatic wavelength of 0.4957 Å (0.4246 Å) and a DECTRIS-PILATUS-3 S 6-M (Rayonix SX165) detector. At Elettra (ALBA) the X-ray beam was focused down and to a spot of 50 μm × 50 μm (20 μm × 20 μm) in size. The sample–detector distance and the detector geometry were calibrated using a LaB_6_ standard and the pressure was measured using the signal of Cu powder loaded together with the sample and the equation of state (EOS) reported by Dewaele et al.^49^ The two-dimensional diffraction images collected with the detectors were processed to create intensity versus 2θ XRD patterns utilizing DIOPTAS.^50^ The analysis of these data was performed by means of Rietveld refinements conducted with the FullProf suite.^51^

The reason for the two HP-XRD measurements was that there were reasonable doubts regarding the nature of the first HP phase above 14 GPa. Initially, we were working with two possibilities (a monoclinic phase and an orthorhombic one). Both were very similar and none of them was theoretically predicted since calculations of enthalpy curves of the two phases were on top of the original *R*3*m* phase. Therefore, we decided to see if a second experiment could give us slightly better results than the first one and clarify which of the two HP candidate phases was the most likely one. At the end, we concluded that the orthorhombic one was the best candidate in reasonable agreement with Raman scattering experiments.

#### 2.2.2 HP-RS Experiments

We performed one HP-RS experiment in powder CsIO_3_ up to 31.2 GPa. RS signal was excited with a 633 nm laser (with a power of less than 10 mW) in backscattering geometry using a Horiba Jobin Yvon LabRAM HR UV microspectrometer. The microspectrometer is equipped with a thermoelectrically cooled multichannel charge-coupled device detector and a 1200 grooves/mm grating that allows a spectral resolution better than 2 cm^–1^. Pressure was determined by the ruby luminescence method.^52^ Raman peaks were analyzed with a Voigt profile fixing the Gaussian line width (1.6 cm^–1^) to the experimental setup resolution.

## 3 Theoretical Details

All theoretical calculations in this paper were performed using density functional theory (DFT) as implemented in the Vienna Ab initio Simulation Package (VASP).^53–56^ The electronic structure was computed using PAW potentials^57^ with Cs (5s^2^, 5p^6^, 6s^1^), I (5s^2^, 5p^5^) and O (2s^2^, 2p^4^) electrons treated as valence electrons. The exchange-correlation term was computed using the PBEsol functional^58^ and the plane waves basis was expanded up to a 560 eV kinetic energy cutoff. The sampling of the Brillouin-zone (BZ) was converged with Γ-centered Monkhorst–Pack^59^ grids employing adequate meshes 8 × 8 × 4 for the trigonal (*R3m*) phase, 6 × 8 × 6 for the orthorhombic (*Pmn2*_*1*_) phase, and 6 × 6 × 8 for the tetragonal (*P*4/*nmm*) phase. The cell parameters and atomic positions for the different structures were fully optimized for a range of volumes by calculating the forces on atoms and the stress tensor. In terms of the resulting optimized configurations, the forces on atoms were smaller than 0.003 eV/Å, and the deviation of the stress tensor components from the diagonal hydrostatic form was less than 0.1 GPa with a convergence of the total energy within 10^–6^ eV.

The phonon properties were computed by using the supercell finite-displacement method implemented in the Phonopy package,^60^ with VASP being used as the second-order force calculator. Supercells were expanded up to 2 × 2 × 2 for both *R3m*, *Pmn2*_*1*_, and *P*4/*nmm* phases, enabling the exact calculation of frequencies at the zone center (Γ) and inequivalent zone-boundary wavevectors, which were then interpolated to obtain phonon-dispersion curves.

A density-based approach grounded in the Quantum Theory of Atoms in Molecules (QTAIM) was employed to analyze the electron density topology of CsIO_3_ in the different phases. Quantum ESPRESSO (version 6.5)^61^ was used for this analysis, in conjunction with Wannier90 ^62,63^ and CRITIC2 programs.^64^ Single-point calculations were performed at the VASP equilibrium geometries, using the same uniform k-point grids mentioned above. A plane-wave cutoff of 100 Ry and a density cutoff of 400 Ry were consistently applied. Norm-conserving pseudopotentials for the Kohn–Sham states and PAW data sets for the all-electron density were sourced from the pslibrary.^65^ Delocalization index (DI) calculations, used to determine the number of electrons shared (ES) between two atoms as 2 × DI, were conducted using a Wannier transformation as described in ref 66. The renormalized number of electrons transferred between two atoms (ET) for I–O bonds was calculated as the Bader charge of the I atom divided by the nominal valence (5+). In addition, this value is divided by 3 to account for the different structural multiplicities of I and O atoms (being the charge of I atoms distributed into 3 O atoms in the IO_3_ units according to the formula unit CsIO_3_) in all phases. All the structures were visualized by VESTA program.^67^ We have to note that our density-based calculations provide a consistent picture of chemical bonding, which is similar to that provided by orbital-based calculations.^15,68^ We have also provided ELF calculations (using VASP) to further analyze the electron density topology. Note that ELF calculations with PAW pseudopotentials using VASP are not all-electron but they contain a partial information on core electrons and have been used in previous works to study phase change materials which have the EDMBs we deal with in this work.^69,70^

## 4 Results and Discussion

### 4.1 Structural Behavior under Compression

XRD patterns of CsIO_3_ at RP (10^–4^ GPa) were assigned to the rhombohedral *R*3*m* structure, as in a previous work.^28^ The assignment is supported by the small residuals of the Rietveld refinements and by the *R*-values obtained (see Figures S1 and S2 in Section 1 in the Supporting Information (SI)), which support a good fit of the structural model to the data (see Table S1 in SI). The *R*3*m* structure can be described as a distorted pseudocubic structure because *c/a* $∼\sqrt{3/2}$ and the structure is composed of CsO_12_ polyhedra and IO_3_ trigonal pyramids with three oxygen atoms in the base and an iodine atom in the vertex. The stereochemically active LEP of iodine points to the opposite direction of the base (along the [001] direction), featuring a well-ordered alignment. Interestingly, a cubic *Pm* $\overset{-}{3}$ *m* structure was identified for CsIO_3_ at RP by Goldschmidt et al. in an early study.^71^ However, this is not the correct structure of our sample since the cubic *Pm*-3*m* structure does not exhibit first-order Raman-active modes while our CsIO_3_ sample exhibits 13 experimental Raman-active modes (see discussion in Section 2 in SI) that are consistent with the assignment to the trigonal structure.

Two HP-XRD experiments at the ALBA and Elettra synchrotron facilities were performed up to a maximum pressure of 40.6 GPa. Both experiments show evidence of a reversible pressure-induced phase transition from the low-pressure (LP) rhombohedral *R*3*m* structure to the HP orthorhombic *Pmn*2_1_ structure (see discussion in Section 1 in SI). Rietveld refinements of XRD patterns above 14 GPa can be better explained with the *Pmn*2_1_ structure than with the *R*3*m* phase (see discussion of Figure S3 in SI). The HP phase can be considered a distorted version of the LP phase. The structural information on the orthorhombic *Pmn*2_1_ structure at 14.4(1) GPa is given in Table S2 in SI. It must be stressed that the existence of the *R*3*m → Pmn*2_1_ phase transition is consistent with the results of our Raman experiments (see the following section) and with the results of our DFT calculations, which show that both phases have the same enthalpy, within the accuracy of calculations, for the pressures of interest for this study.^72^

From the XRD patterns, we obtained the pressure dependence of the unit-cell parameters (Figure 2a) and unit-cell volume (Figure 2b) of the two phases of CsIO_3_. The compression is anisotropic, being the *c*-axis more compressible than the *a*-axis in the hexagonal unit-cell of the *R*3*m* phase. This explains the peak splitting we described when discussing Figures S1 and S2 in SI. The anisotropic behavior of the structure can be better appreciated in the decrease of the *c/a* ratio at HP and its deviation from the $\sqrt{3/2}$ value (see inset of Figure 2b). Figure 2b shows that our calculations reproduce the behavior of the experimental *c*/*a* ratio of the *R*3*m* phase and the unit-cell parameters of the *R*3*m* and *Pmn*2_1_ phases, which point to the convergence of the *a* and *c* lattice parameters of the HP phase above 45 GPa. This result confirms the hypothesis of the expected PIS of the structure of CsIO_3_. Data of the experimental and theoretical equation of state (EOS) of CsIO_3_ under compression are provided in Section 1 in SI. It must be stressed that CsIO_3_ is one of the most compressible iodates,^44^ indeed as compressible as Sr­(IO_3_)_2_HIO_3_ ^41^ and as KIO_3_.^37^

**Figure 2:**
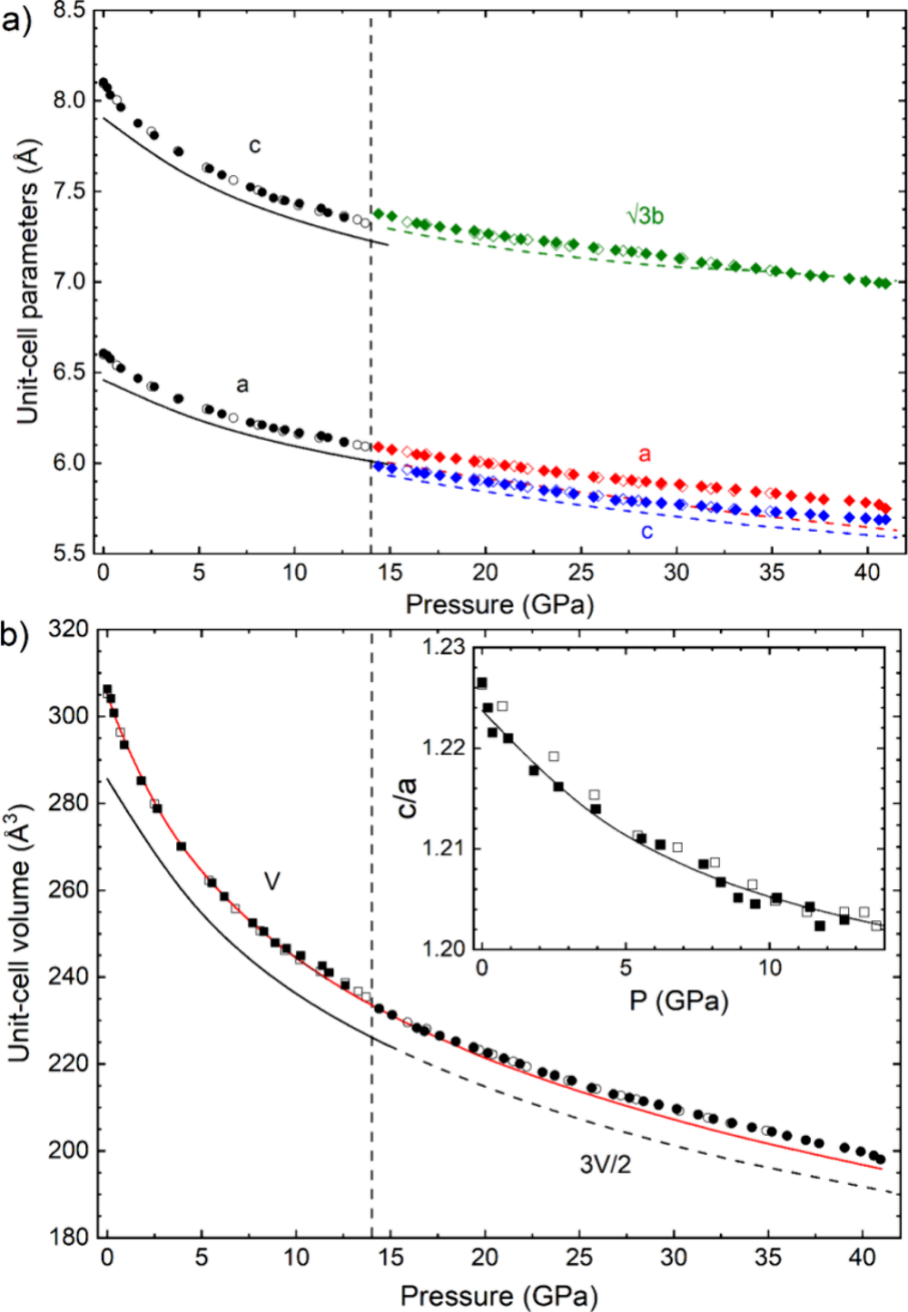
Pressure dependence of the unit-cell parameters (a) and volume (b) of the LP *R*3*m* and HP *Pmn*2_1_ phases. Solid (empty) symbols are used for the ALBA (Elettra) experiments. Black (color) symbols are used for the LP (HP) phase. Solid (dashed) lines correspond to results from DFT calculations for the LP (HP) phase. Unit-cell parameters are identified in the figure. For the HP phase, we have plotted $\sqrt{3}$ *b* to facilitate the comparison with the LP phase. The red line is the experimental EOS described in the text. The unit-cell volume of the HP phase was renormalized to facilitate the comparison with the LP phase. The inset shows the pressure dependence of the *c/a* ratio in the LP phase.

Rietveld refinement has allowed us to obtain the evolution of the experimental atomic parameters of both *R*3*m* and *Pmn*2_1_ phases of CsIO_3_ up to 40 GPa. As observed in Figure S4 in SI, there is a nice agreement between experimental and theoretical atomic parameters in the *Pmn*2_1_ phase from 14.4 to 40 GPa. Importantly, our theoretical calculations up to 70 GPa show that the atomic parameters of the *Pmn*2_1_ phase evolve toward values compatible with a tetragonal phase (SG *P*4/*nmm*, No. 129) above 45 GPa. The structural parameters of the *P*4/*nmm* phase at 52 GPa are summarized in Table S3 in SI. Figure 3 shows a picture of the tetragonal *P*4/*nmm* phase of CsIO_3_ expected above 45 GPa and a comparison with the orthorhombic *Pmn*2_1_ phase.

**Figure 3:**
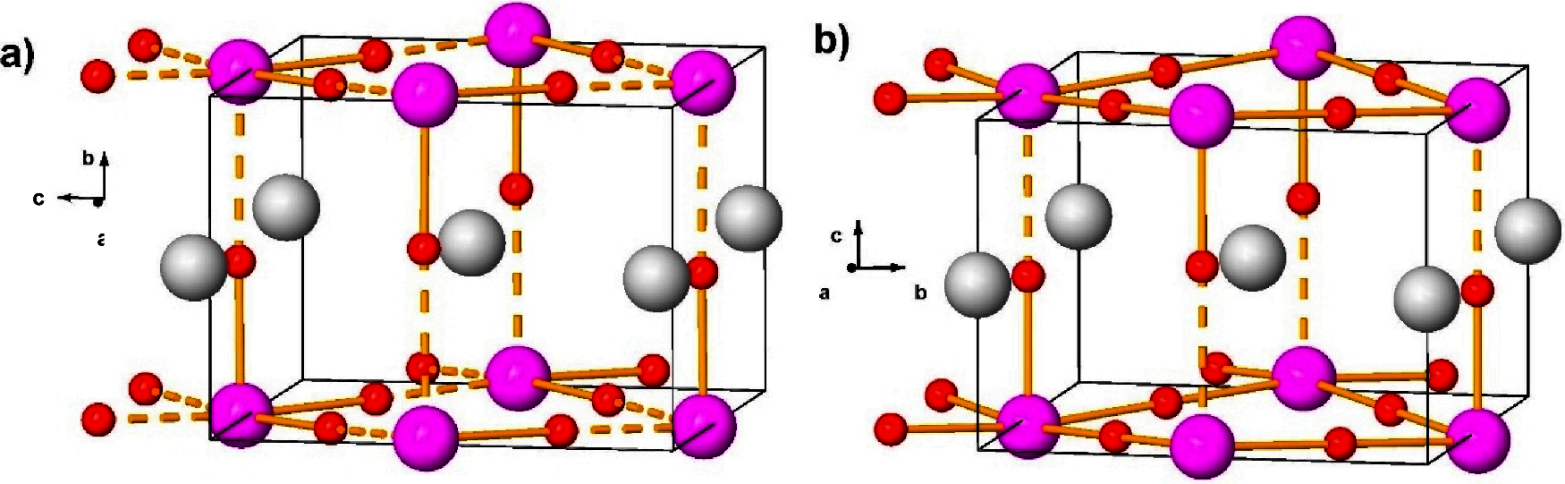
Crystal structure of CsIO_3_ in (a) the HP orthorhombic *Pmn*2_1_ phase at 14.4 GPa (see data of Table S2) and (b) the HP tetragonal *P*4/*nmm* phase at 52 GPa (see data of Table S3). The perspective of both structures has been selected to highlight their similitudes. The orthorhombic phase at 14.4 GPa still exhibits IO_3_ trigonal pyramidal units since there are very distorted IO_6_ units with three long I···O bond distances (dashed orange lines) along the *a*, *b,* and *c* axes. The tetragonal phase still exhibits slightly distorted IO_6_ octahedra that can be described as square pyramidal IO_5+1_ units since there is a long I···O distance (dashed orange line) along the *c*-axis. Note that all I–O distances in the *ab* plane of the tetragonal phase are equal and slightly larger than the short I–O bond along the *c*-axis; therefore, the tetragonal structure is a slightly distorted (quasi-cubic) perovskite structure.

From the experimental and theoretical pressure dependence of the atomic parameters, we have calculated the pressure dependence of the experimental and theoretical I–O bond lengths in the three phases (*R*3*m*, *Pmn*2_1_, and *P*4/*nmm*) of CsIO_3_ (Figure 4a). As observed, the 3-fold-degenerated short delocalized resonant I–O bond of the LP phase (intramolecular bonds inside the IO_3_ units) shows a slight increase in bond length with increasing pressure up to 14 GPa, while the 3-fold-degenerated long secondary noncovalent I···O bond of the LP phase (intermolecular bonds between neighbouring IO_3_ units) show a considerable decrease (14%) in bond length in the same pressure range; i.e., an astonishing average change of around 1% per GPa. Therefore, IO_3_ units exhibit a strong tendency to form IO_6_ units, as would be expected in a cubic or slightly distorted PV structure. The increase in symmetry of the IO_6_ units (as all the I–O bonds tend to equalize) is not completed at 14 GPa and continues above this pressure in the *Pmn*2_1_ phase. In this HP phase, the 3-fold-degenerated short and long I–O bond lengths (six in total) of the LP phase split into one single and one 2-fold-degenerated short and long I–O bond distances (six in total). The single short and long I–O bond lengths (two in total) are along the *b-*axis of the orthorhombic phase, while the 2-fold-degenerate short and long I–O bond lengths (four in total) are in the *ac-*plane of the orthorhombic phase. Interestingly, the 2-fold-degenerated short and long distances in the *ac-*plane tend to become equal as pressure increases; i.e., four of the original six bonds in the LP phase tend to the same bond length in the HP *Pmn*2_1_ phase. Our calculations, which nicely reproduce the experimental results, predict that the mentioned PIS occurs beyond 45 GPa once the HP tetragonal *P*4/*nmm* phase is formed (see black dashed lines in Figure 4a). On the contrary, the single short I–O and long I···O bond distances along the *b*-axis of the *Pmn*2_1_ phase, which tend to equalize in the pressure range between 14 and 25 GPa, display an opposite tendency above 25 GPa and remain as short and long bonds in the *P*4/*nmm* phase (see squares and solid lines in Figure 4a). This results in the formation of IO_5+1_ units in the tetragonal *P*4/*nmm* phase as shown in Figure 3b.

**Figure 4:**
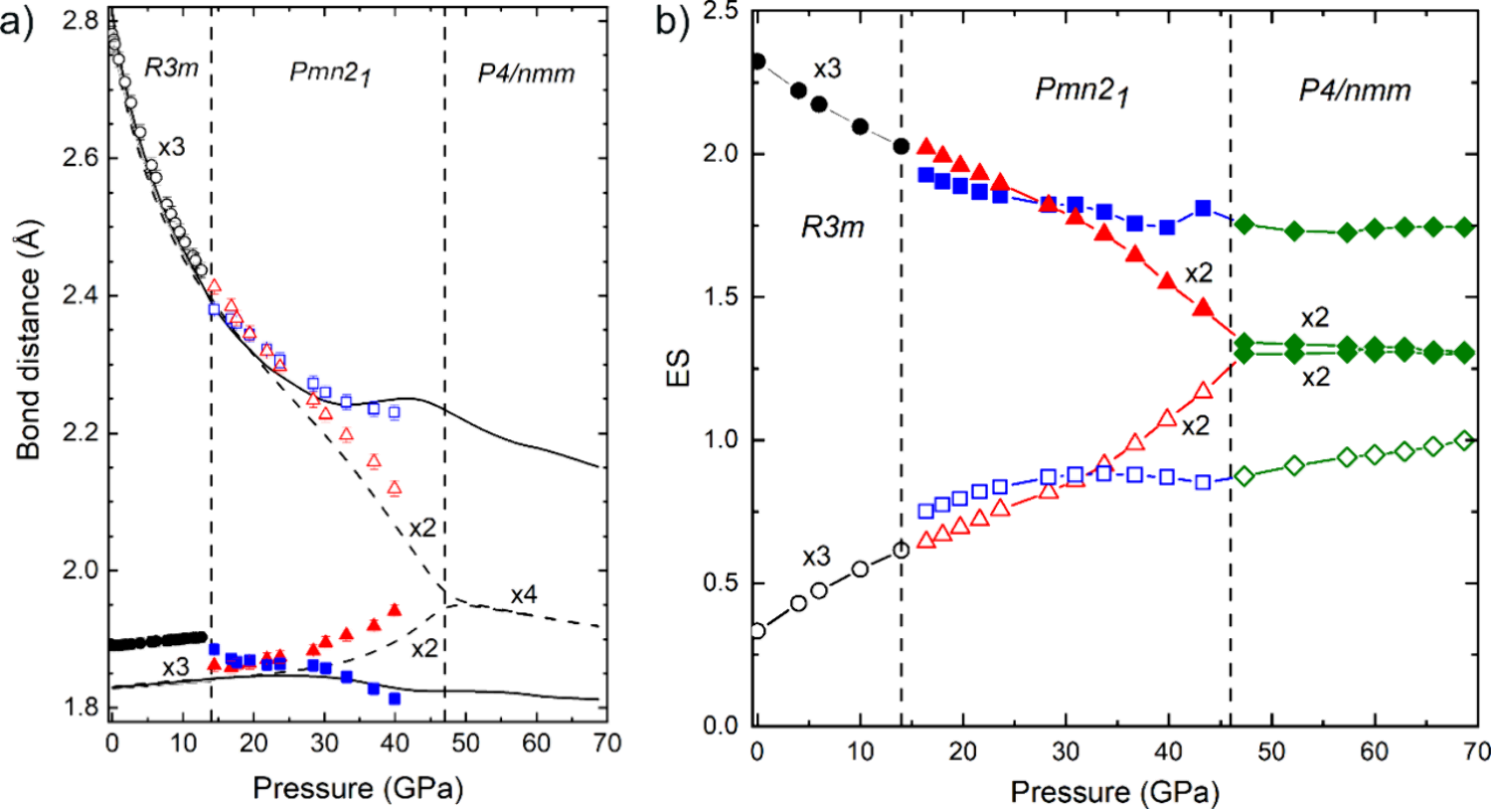
(a) Pressure dependence of experimental (symbols) and theoretical (lines) I–O bond distances in the different phases of CsIO_3_. Circles (triangles and squares) correspond to the *R*3*m* (*Pmn*2_1_) phase. Solid (open) symbols correspond to the short and long distances in both *R*3*m* and *Pmn*2_1_ phases. Solid and dashed lines correspond to theoretical bonds with different pressure behavior. Dashed vertical lines indicate the pressures for the experimental (first) and theoretical (second) phase transitions. (b) Pressure dependence of theoretical ES values of the I–O bond distances in the different phases of CsIO_3_. To help in the interpretation of the theoretical data we have used the same symbols as in (a) for the *R*3*m* and *Pmn*2_1_ phases. For the *P*4/*nmm* phase solid (open) green diamonds are plotted to distinguish between short (long) bonds.

As commented in the Introduction, the PIS of the crystalline structure of CsIO_3_ was expected due to a theoretical work in isostructural RbIO_3_ which suggested that the full PIS would end with the formation of the cubic PV structure (*Pm*-3*m*, S.G. No. 221) around 35 GPa.^24^ Instead, our calculations predicted that a full PIS of the structure in CsIO_3_ does not occur even at 70 GPa, although a full PIS at higher pressures cannot be discarded.

In summary, experimental and theoretical structural information in CsIO_3_ under compression suggests the presence of two pressure-induced phase transitions up to 50 GPa, the first one (from rhombohedral to orthorhombic) around 14 GPa and the second one (from orthorhombic to tetragonal) above 45 GPa. The transitions occur without appreciable changes in the unit-cell volume; however, it does not exist a group-subgroup relationship either between the *R*3*m* and *Pmn*2_1_ space groups or between the *Pmn*2_1_ and *P*_4_/*nmm* space groups; thus indicating that the transitions could not be of second order^73^ and are likely weak first-order transitions. The fact that there is no bond breaking/formation at the transitions supports a displacive mechanism for these transitions, which is consistent with their reversibility.^74^ These phases occur before the possible phase transition toward the cubic PV phase above 70 GPa. It must be noted that the PIS following the rhombohedral-orthorhombic-tetragonal-(cubic) sequence of phase transitions in CsIO_3_ agrees with that expected for the formation of the cubic PV structure, as observed in BaTiO_3_ with increasing temperature (see Figure 6.2 in ref 4) and also similar (in this case the rhombohedral-orthorhombic phases are reversed) to that predicted for LaGaO_3_ under compression.^75^ We have also to note that the rhombohedral-to-orthorhombic phase transition near 14 GPa is perhaps induced by non-hydrostatic conditions since our calculations of both phases on hydrostatic conditions indicate that both phases have the same enthalpy in the whole pressure range studied.^72^

The orthorhombic-tetragonal phase transition above 45 GPa is a phase transition resulting in a PIS of the IO_6_ octahedra which leads to a 5 + 1 coordination for I atoms; i.e., IO_5+1_ units are formed in the tetragonal PV phase. The slightly distorted PV structure of the tetragonal phase in CsIO_3_ at 52 GPa can be noted if we compare the I–O bond distances in IO_5+1_ units (with one I–O bond at 1.824 Å, four at 1.948 Å, and one much longer at 2.205 Å) with those of the TiO_5+1_ units present in the tetragonal distorted PV phase of BaTiO_3_ (with one Ti–O bond at 1.86 Å, four at 2.00 Å and one longer at 2.17 Å) as reported at page 469 in ref 3. Similar *B*O_5+1_ units are also present in other tetragonal PV phases, as that of T*-Nd_2–*y*–*z*_Ce_*y*_Sr_*z*_CuO_4_ (see Figure 6.10 in ref 4).

### 4.2 Vibrational Behavior under Compression

A selection of the measured RS spectra of CsIO_3_ at different pressures is shown in Figure S6 in SI. A total of 13 Raman modes have been experimentally detected in the range up to 1000 cm^–1^, in agreement with those previously reported at RP.^76^ These results are consistent with group theory predictions for the rhombohedral *R*3*m* phase. The different Raman-active modes observed in the LP phase are provided in Table S4 in SI and discussed in Section 2 in SI. Modes of the LP phase are observed up to 16.4 GPa and changes in the Raman spectrum are observed between 13.5 and 16.4 GPa (see Figure S6), in agreement with the phase transition detected by HP-XRD experiments. The increase in the number of Raman modes observed above 13.5 GPa is consistent with a decrease in the symmetry of the crystal structure at the phase transition. Again, this is in agreement with what is observed in HP-XRD experiments.

According to group theory,^77^ the orthorhombic *Pmn*2_1_ phase (SG No. 31, *Z* = 2) of CsIO_3_ has 30 vibrational modes, comprising 3 acoustic modes (A_1_+ B_1_+ B_2_) and 27 optical modes (8A_1_ + 6A_2_ + 5B_1_ + 8B_2_). All optical modes are both Raman- and infrared (IR)-active except for the A_2_ modes, which are silent ones. This means that both the A_1_, B_1_, and B_2_ modes exhibit TO-LO splitting. Therefore, a total of 42 Raman- and IR-active modes are expected in the *Pmn*2_1_ phase of CsIO_3_. In contrast, in our RS measurements, we have only detected a total of 13 Raman modes.

Figure 5 shows the experimental and theoretical pressure dependence of the wavenumber of the Raman-active modes in both *R*3*m* and *Pmn*2_1_ phases of CsIO_3_ up to 30.2 GPa. Experimental data (symbols) are compared to theoretical calculations (lines) which include the TO-LO splitting. Our calculations show that while TO-LO splitting is considerable for E modes, the splitting is negligible for A modes. In the *R*3*m* phase, there is a good agreement between experiments and calculations both for the wavenumbers of the first-order Raman-active modes (within 5% accuracy) and their pressure dependence. While the assignment of the symmetries of the Raman-active modes of the LP phase is relatively easy (see discussion in Section 2 in SI), the symmetries of the experimental Raman-active modes of the HP phase of CsIO_3_ are considerably more difficult to assign (see discussion in Section 2 in SI where a tentative assignment is proposed in Table S5 in SI). In any case, the number of Raman modes and their pressure dependence is consistent with the *Pmn*2_1_ phase at least between 15 and 25 GPa.

**Figure 5:**
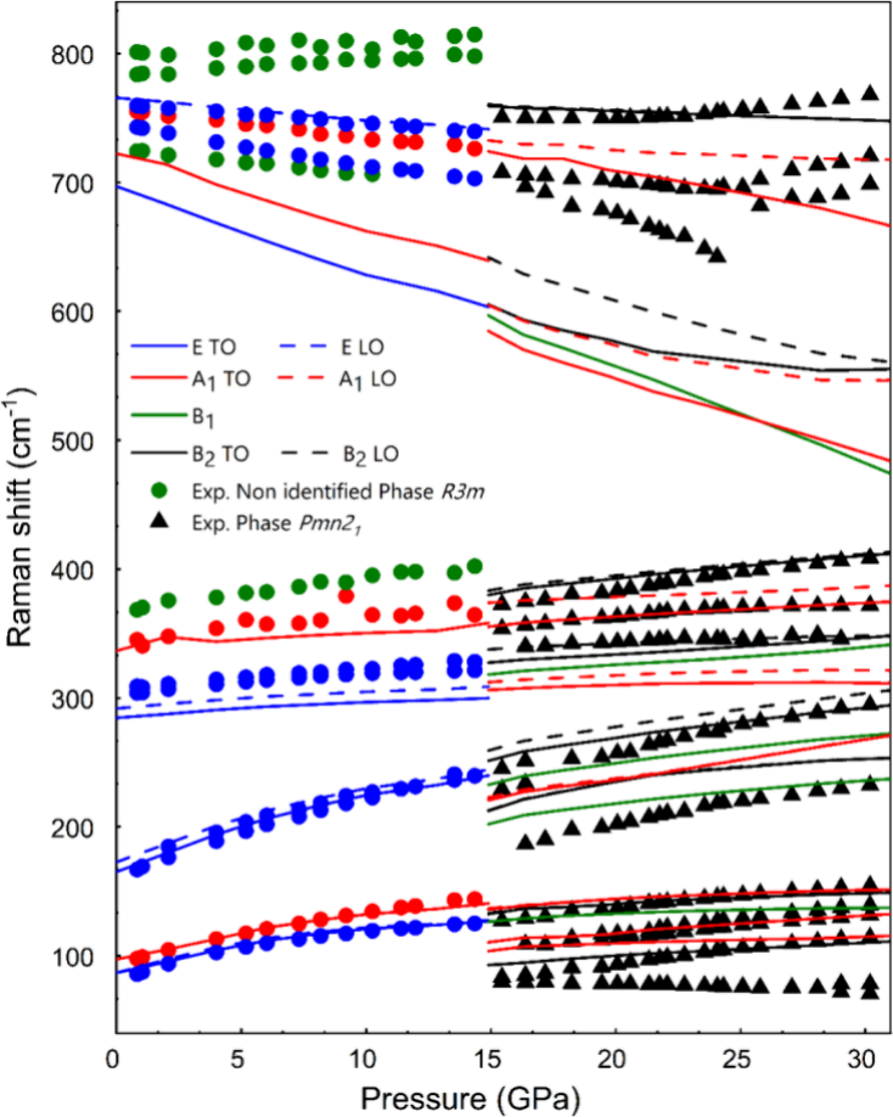
Pressure dependence of the Raman modes of CsIO_3_ up to 30.2 GPa. Symbols (lines) correspond to results from experiments (DFT calculations). Circles (triangles) correspond to the *R*3*m* (*Pmn*2_1_) phase. Up to 15 GPa, red and blue colors correspond to A_1_ and E modes of the *R*3*m* phase, respectively, while the green color represents modes that likely are not first-order modes. Above 15 GPa, red, green, and black colors correspond to A_1_, B_1_, and B_2_ modes of the *Pmn*2_1_ phase. Solid lines correspond to TO phonons while dashed lines correspond to the LO phonons. Note that LO phonons of A_1_ modes in the *R*3*m* phase and of B_1_ modes in the *Pmn*2_1_ phase are not observed, especially at low wavenumbers, due to the negligible LO-TO splitting.

The most interesting result for our purpose is the observed softening of the high-wavenumber modes in both *R*3*m* and *Pmn*2_1_ phases of CsIO_3_, at least up to 25 GPa, which leads to the almost complete closing of the phonon gap between the stretching and bending modes of the IO_3_ units at 30 GPa. Noteworthy, the negative pressure coefficient of the high-wavenumber stretching modes in iodates has been already reported and explained by the increase of the short I–O bond distance and the loss of charge of these short primary delocalized resonant (covalent-like) I–O bonds.^38–41^ Also noteworthy, the soft phonon behavior upon approaching the cubic PV phase is consistent with what has been observed in many compounds.^5^

### 4.3 Chemical Bonding under Compression

The loss of charge of the short primary I–O bonds in both *R*3*m* and *Pmn*2_1_ phases of CsIO_3_ indicated by the softening of the high-wavenumber modes in both phases suggests a pressure-induced change in chemical bonding in this compound. The change in chemical bonding is confirmed by the decrease in the number of electrons shared (ES) between two atoms (see theoretical details in Section 3 in the SI), according to QTAIM,^16^ plotted in Figure 4b. The ES values of the short I–O bonds in IO_3_^–^ units of the *R*3*m* phase are larger than 2 at RP. This value is consistent with the delocalized resonant (covalent-like) character of the I–O bonds in IO_3_^–^ units. The delocalized resonant bonding in IO_3_^–^ units is similar to that of well-known NO_3_^–^ units, with the difference that there is a resonance between one single covalent bond and two double covalent bonds in IO_3_^–^ units and a resonance between two single covalent bonds and one double covalent bond in the NO_3_^–^ units due to the larger number of valence electrons in halogens than in pnictogens.

The delocalized resonant I–O bonding in IO_3_^–^ units is also consistent with the I–O bond lengths. Notice that the bonds in the IO_3_^–^ units in CsIO_3_ have bond lengths of around 1.9 Å at RP. This value is intermediate between the larger single covalent I–O bond and the shorter double covalent I–O bond, estimated to be 1.96 and 1.86 Å from the covalent radii, respectively.^78,79^ These values are in turn consistent with reported values in other iodates.^42,43,80–84^

Interestingly, the ES values of the short primary I–O bonds decrease at the same rate as the ES values of the long secondary I···O bonds increase in both *R*3*m* and *Pmn*2_1_ phases. This concomitant ES decrease of the short bonds and the ES increase of the long bonds can be interpreted as a multicenter interaction in which there is a charge transfer from the primary, short I–O bonds toward the secondary, long I···O bonds. The charge transfer is a consequence of the *trans influence* of the secondary bond into the primary bond that occurs as a prior step (stage 2 of 3) to the formation of multicenter bonds (stage 3) in the process of formation of multicenter bonds according to the recent unified theory of multicenter bonding.^14,15^

According to the mentioned theory, the charge transfer ends once the two I–O bond types (short and long) equalize and form the multicenter bonds (stage 3).^14,15^ From that point on, all I–O bond lengths tend to decrease with pressure, and the soft high-wavenumber modes disappear; i.e., high-wavenumber vibrational modes (related to stretching vibrations) show a positive pressure coefficient. This is exactly what happens once the *P*4/*nmm* phase crystallizes above 45 GPa, as shown by our theoretical simulations (see Figure S7 in SI).

According to our ES calculations, the formation of the tetragonal *P*4/*nmm* phase above 45 GPa leads to the formation of 2D electron-deficient multicenter bonds (EDMBs) in the *ab* plane of the tetragonal PV structure (see Figure 3b) that come from the equalization of short and long bonds in the *ac* plane of the orthorhombic PV structure (see Figure 3a). The EDMBs are characterized by ES values close to 1, unlike the ERMBs, which typically show values of ES well above 1.4.^14,15^ In the case of the I–O EDMBs within the IO_5+1_ units of the *P*4/*nmm* phase, the ES value is ca. 1.25 because of the partial resonant bonding still present in this phase. As already explained, this resonant bonding explains why the I–O bonds within the IO_3_ units of the *R*3*m* phase have ES values well above 2 (typical of resonant bonds). In addition to the two EDMBs, there are two additional bonds along the *c*-axis in the IO_5+1_ units of the *P*4/*nmm* phase. The fifth I–O bond, which is the shortest I–O bond, still preserves part of the delocalized resonant character already present at the LP phase, while the sixth I–O bond is the longest one and still preserves a partial noncovalent character similar to those of the LP phase. Note that the bond length of the four I–O EDMBs in the *ab* plane of the *P*4/*nmm* phase in CsIO_3_ is ca. 1.95 Å, which is similar to the value of the long bonds present in I_2_O_5_ units^83^ and I_4_O_12_ units.^84^ This suggests the possibility of observation of EDMBs in some iodates at RP; a feature worthy to be studied in future works.

The change in the I–O bonds in CsIO_3_ across its successive pressure-induced crystalline phase transitions can be better understood by the analysis of the electron localization function (ELF). At the left of Figure 6a–d, we show the ELF isosurfaces in the three crystalline structures at four different pressures, while at the right of Figure 6e–h we show the corresponding ELF values along the different I–O bonds present in each phase. In the *R*3*m* phase at 0 GPa, the I atom shows a basin (corresponding to the iodine LEP) oriented along the axis perpendicular to the plane defined by the three O atoms of the IO_3_ units. At this pressure, the O atoms show a basin which is intermediate between a toroidal and quasi-circular basin around each O atom (corresponding to the electrons forming part of the LEPs and the delocalized electrons of the resonant bonding). From the point of view of the IO_6_ units, the *R*3*m* phase is characterized by two different bonds, the short primary 3-fold degenerated intramolecular I–O bonds of the IO_3_ units and the long secondary 3-fold degenerated intermolecular I–O interactions between the IO_3_ units. In Figure 6e, the ELF values of the short primary I–O bonds at RP show a weak minimum between I and O atoms (with a value of ca. 0.7 around the value of 0.5 for the normalized distance). This shape of the ELF profile (without a well-localized minimun) is not expected for a covalent bond (with a well-localized minimum) and is consistent with the delocalized resonant (covalent-like) character of the I–O bonds in the *R*3*m* phase at RP. On the other hand, the ELF value of the long secondary I–O interactions exhibits a marked minimum between I and O atoms (around 0.56) whose value close to 0.1 evidences the weak noncovalent character of the secondary interaction at RP.

**Figure 6:**
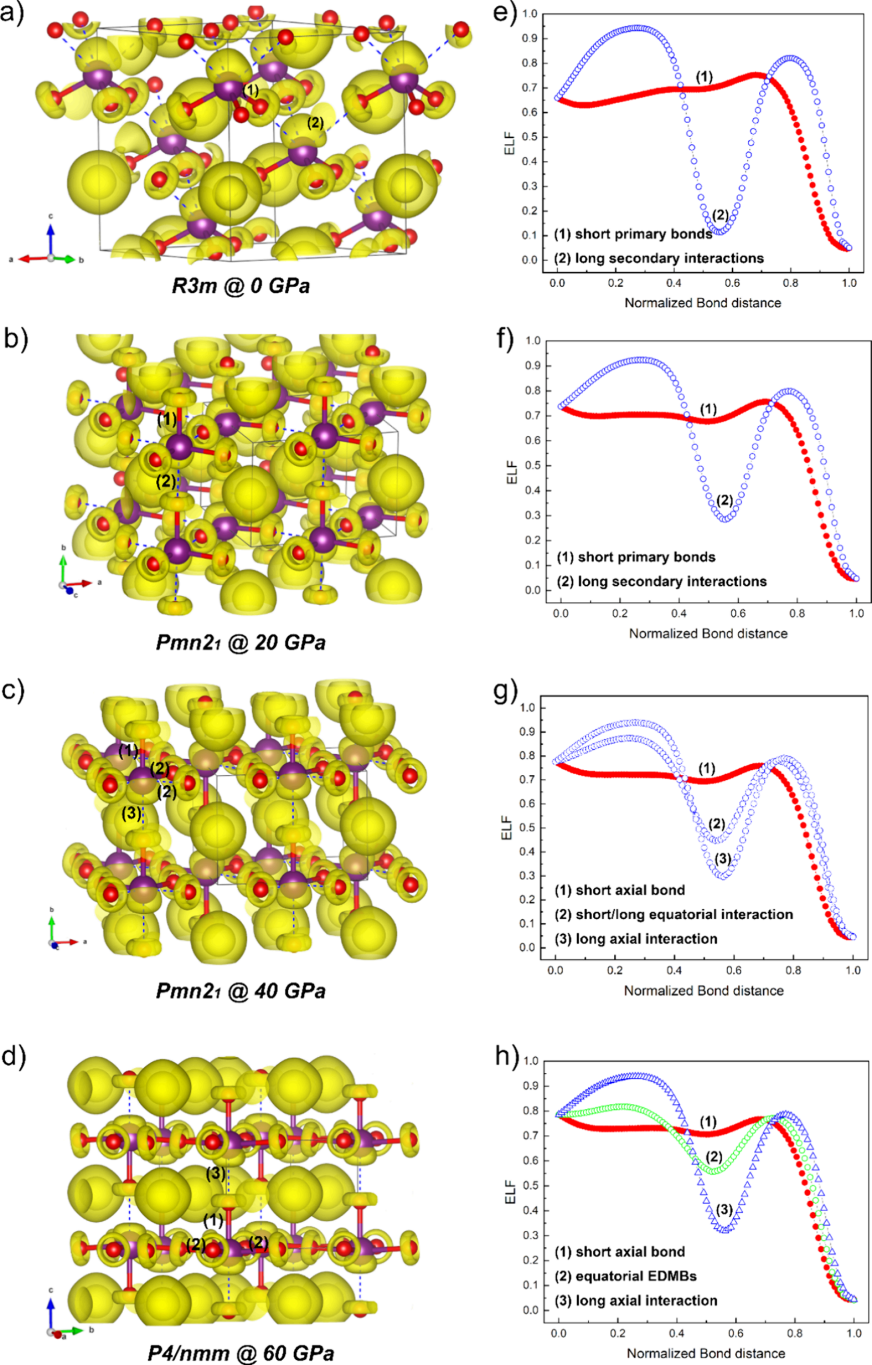
Electron localization function (ELF) isosurfaces and ELF values along the different I–O bonds of CsIO_3_ for the *R*3*m* phase at 0 GPa (a, e), the *Pmn*2_1_ at 20 GPa (b, f) and 40 GPa (c, g), and the *P*4/*nmm* phase at 60 GPa (d,h). In (a), (b), (c), and (d), yellow isosurfaces correspond to the lone electron pairs (LEP) around I and O atoms, the short bonds are depicted with wide bars in red and magenta colors, and the long bonds are indicated with dashed blue lines.

In the *Pmn*2_1_ phase at 20 GPa, the I atom shows a LEP is still oriented as in the *R*3*m* phase (Figure 6b), while all O atoms show more defined toroidal basins oriented perpendicular to the more defined linear I–O–I bonds (the I–O–I angle changes from 168° at RP to 175° at 20 GPa). From the point of view of the IO_6_ units, there are four different I–O bonds in this phase, the short and long bonds along the *b* axis and the 2-fold degenerate short and long bonds in the *ac* plane; however, the difference between the short/long bonds along the *b* axis and in the *ac* plane cannot be distinguished (see Figure 4a). Consequently, they show ELF values (Figure 6f) similar to those of the *R*3*m* phase, with the difference that the long bonds are stronger than at RP (the minimum of the ELF changes from ca. 0.1 at RP to ca. 0.3 at 20 GPa). In the *Pmn*2_1_ phase at 40 GPa, the I atom shows a LEP reoriented along the secondary I–O bond in the *b*-axis, while the LEPs of all O atoms in the *ac* plane show almost a perfect toroidal form (Figure 6c). From the point of view of the IO_6_ units, there are four different I–O bonds in this phase at 40 GPa, the short and long bonds along the *b* axis and the 2-fold degenerate short and long bonds in the *ac* plane. The ELF value of the short and long bonds along the *b* axis (axial bonds) still show the same resonant delocalized and noncovalent characters as at 20 GPa, respectively (see red and blue symbols in Figure 6g). On the other hand, the short and long bonds in the *ac* plane (equatorial bonds) show ELF values that tend to approach each other; i.e., there appears a minimum in the short bonds (consistent with a weakening of the intramolecular bonds), and the ELF value at the minimum of the long bonds increases (showing a strengthening of the intermolecular bonds).

Finally, in the *P*4/*nmm* phase at 60 GPa, the I atom still shows a LEP oriented along the secondary I–O bond in the *c*-axis, while the LEPs of all O atoms show a toroidal basin (Figure 6d). The toroidal basins are totally symmetric (corresponding to the six electrons of three LEPs) for the O atoms located in the *ab* plane, while are asymmetric for the O atoms along the *c* axis. This can be explained from the point of view of the IO_6_ units because there are three different I–O bonds in this phase, the short and long bonds along the *c-*axis and the 4-fold degenerate bond in the *ab-*plane (see Figure 4a). The ELF value of the short and long bonds along the *c-*axis still shows the same delocalized resonant and noncovalent character as at 0 GPa, respectively, although the strength of the secondary bond has notably increased. Interestingly, the four-degenerate bond in the *ab-*plane shows intermediate ELF values between those of the primary delocalized resonant bonds and the secondary noncovalent bonds (Figure 6h). These ELF values correspond to the EDMBs formed in the *ab* plane; therefore, it can be speculated that these are the ELF values that will be reached by all six I–O bonds (forming three EDMBs along the three directions of space) in the regular IO_6_ unit of the cubic PV phase of CsIO_3_ once PIS takes place along the *c-*axis of the tetragonal phase at much higher pressures. In fact, an almost total PIS has been found in our calculations for RbIO_3_ (see Figure S8 in SI). This compound goes from the *R*3*m* phase at RP toward the quasi-cubic *R*-3*m* phase at 60 GPa. At that pressure, all bonds are equal up to the fourth decimal place, so the simulated phase can be considered as a very subtle distortion of the cubic *Pm*-3*m* phase. In RbIO_3_, the almost total PIS leads to the formation of EDMBs in all three directions, so the LEP corresponding to the I atom is now symmetrically distributed around I atoms as it corresponds to an inactive LEP, whose (s-type) electrons do not participate in bonding. At the same time, the LEPs of O atoms along the three spatial directions show perfectly symmetric toroidal basins in the plane perpendicular to the I–O–I bond lines..

It must stressed that square pyramidal PbBr_5_ units, similar to the units found in the *P*4/*nmm* phase of CsIO_3_, have been recently found in 1D chains of lead bromide at RP.^85^ The Pb–Br bonds in the square basis of the pyramidal unit are longer (average 3.04 Å) than the Pb–Br bond perpendicular to the square basis (2.71 Å). This suggests that this last bond is a covalent bond, while those of the square basis are EDMBs similar to those found in the PbBr_6_ units of the *Pnma* phase in the distorted PV RbPbBr_3_ (average ca. 3.05 Å).^86^

The pressure-induced evolution of the I–O bonds in the distorted PV CsIO_3_ across its successive crystalline phase transitions can be monitored using the ES vs ET map (Figure 7). For this purpose, the Bader charges, along with the ES and ET values for the short I–O bonds in various phases and pressures are provided in Table S6 in SI. In the rhombohedral *R*3*m* phase, the three short I–O bonds at 0 GPa are located slightly above the red region, indicating their delocalized resonant (covalent-like) character. In the orthorhombic *Pmn*2_1_ phase, the short I–O bonds remain within the red region but shift closer to the green region (where EDMBs are located) as pressure increases (see different values for the three short bonds at 20 and 40 GPa). In the tetragonal *P4/nmm* phase at 60 GPa, the ES values of the two types of short I–O bonds behave differently: the bond along the *c* axis remains in the red region, while those in the *ab* plane move into the green region. This means that the short I–O bonds in the *ab* plane within the IO_5+1_ octahedra are no longer covalent bonds and have transformed into EDMBs.

**Figure 7:**
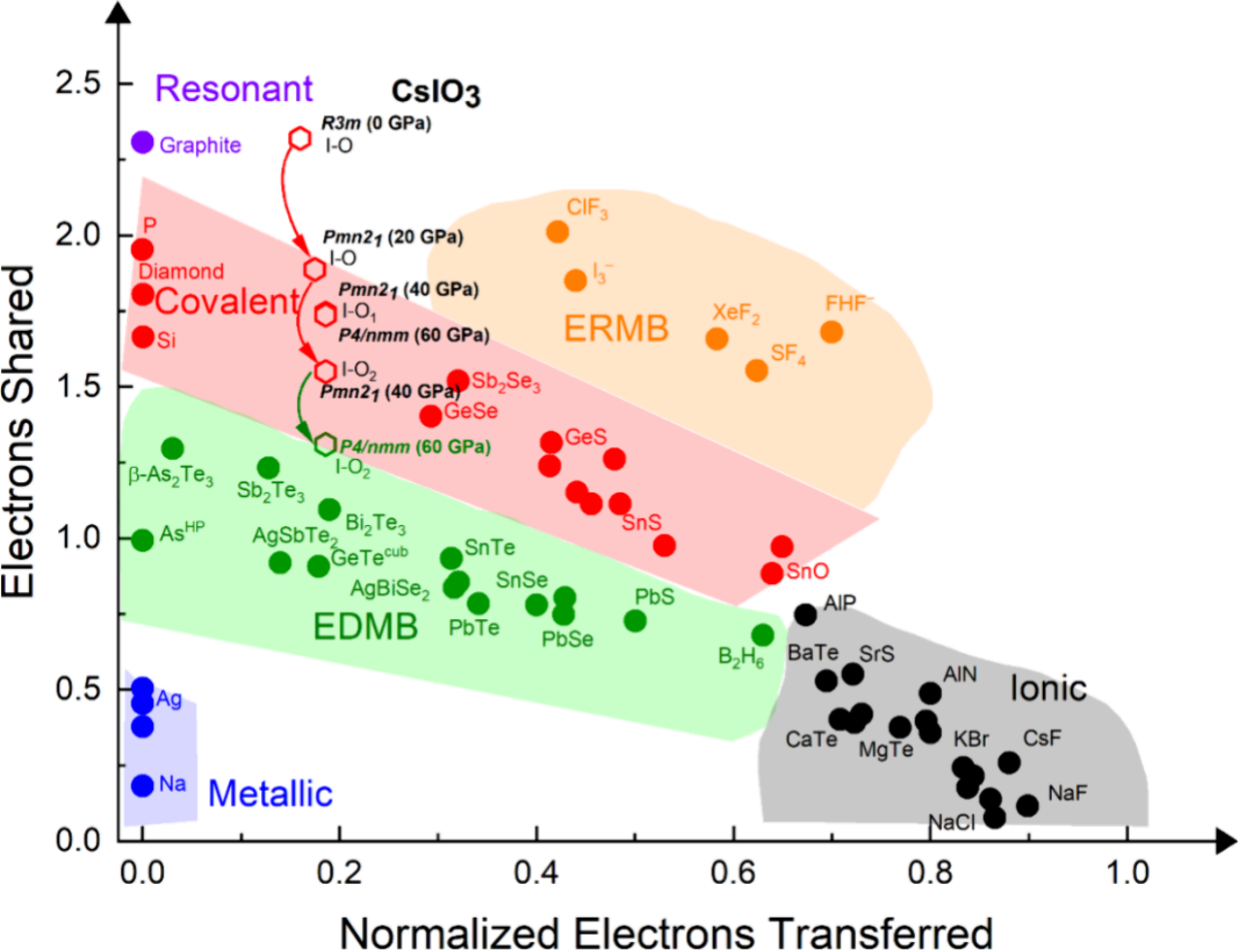
2D map of the number of electrons shared (ES) vs the normalized number of electrons transferred (ET). The ES and ET values of the I–O bonds in the different phases of CsIO_3_ at different pressures are indicated. When several similar bonds are present the average ES and ET values are represented.

The trend of chemical bonds in CsIO_3_ and RbIO_3_ under compression shown in Figures 6, 7 and S8 suggests that in the cubic *Pm-3m* phase of *A*IO_3_ compounds the ES value of all I–O bonds would be the same, and similar to those already present in the *ab* plane in the tetragonal PV phase of CsIO_3_. In other words, all I–O bonds in the regular IO_6_ octahedra in the hypothetical cubic PV phase would be EDMBs. This result is confirmed in our calculations of the ES values of the quasi-cubic *R*-3*m* phase of RbIO_3_ at 60 GPa (see Table S7 in SI). Note that this result is consistent with the claim of Wuttig and co-workers, who found metavalent (electron-deficient) bonds in the simulated cubic PV structure of CsPbX_3_ (X = F, Cl, Br, I) lead halides.^11^ In this regard, we recall that we consider that metavalent bonds do not exist since they are linear (or quasi-linear) combinations of concatenated 3c–2e EDMBs, as explained in the unified theory of multicenter bonding.^14,15^

The unified theory of multicenter bonding allows us to explain the tendency to equalization of short and long I–O bonds (Figure 2a) and the softening of the high-wavenumber vibrational modes (Figure 5) in the *R*3*m* and *Pmn*2_1_ phases of CsIO_3_ since there is a multicenter interaction taking place in the whole pressure range from RP to 45 GPa. In fact, the decrease of all bonds (Figure 4a) and the hardening of the high-wavenumber vibrational modes (Figure S7) above 50 GPa indicates that this multicenter interaction finishes above 45 GPa when EDMBs are formed in the *ab* plane of the tetragonal phase (stage 3). Note that the charge transfer from the short I–O bonds toward the long I···O bonds, shown in Figure 4b, indicates that the multicenter bonds have an electron-deficient character as suggested by the multicenter bonding theory.^14,15^

We have also to note that the multicenter interaction should persist along the *c*-axis of the tetragonal phase as pressure increases beyond 50 GPa because the PIS is not completed along this axis; however, our calculations show that the multicenter interaction is no longer present in the *P*4*/nmm* phase of CsIO_3_. We may speculate that the lack of *trans influence* of the secondary bond (along the *c*-axis of the tetragonal phase) into the primary bond (along the same *c*-axis) is caused by steric effects related to the large volume of the Cs atom that avoids further contraction of the structure along the *c*-axis. Note that such an effect is not observed in RbIO_3_ (Figure S8). In fact, the lack of multicenter interaction along the *c*-axis in the *P*4*/nmm* phase of CsIO_3_ is already observed along the *b*-axis of the *Pmn*2_1_ phase above 20–25 GPa. Above this pressure range, it occurs the separation of the degenerated short and long bonds into the bonds of the *ac* plane (that will become EDMBs) and the short and long bonds along the *b*-axis (that will remain as covalent and noncovalent interactions, respectively). This separation leads to a contraction of the short bond along the *b* axis (see blue squares in Figure 4a) resulting in an upturn in the corresponding vibrational modes, which show a sudden increase in wavenumber (see experimental and theoretical vibrational modes around 700 and 600 cm^–1^, respectively, in Figures 5 and S7). Moreover, this contraction of the short bond along the *b*-axis of the *Pmn*2_1_ phase is consistent with the expected contraction of the delocalized resonant I–O bond (see red line in Figure S5 in SI whose extrapolation to RP leads to a value close to 1.90 Å as the I–O bonds at RP).

### 4.4 Electronic Behavior under Compression

It is noteworthy that the formation of EDMBs in the tetragonal PV phase of CsIO_3_ is consistent with a pressure-induced change in the simulated electronic properties of the distorted PV (Figure 8). In particular, an increase of the average dielectric constant, ε, and the Born effective charges, Z*, are observed due to the pressure-induced “metallization”, related to the closing of the bandgap, taking place between 40 and 50 GPa. Note that our calculations, which underestimate the bandgap, predict the closing around 43 GPa, so the experimental bandgap closure is expected at higher pressures. A similar evolution of these properties was observed in pnictogens, chalcogens, and related phase change materials under compression upon the formation of EDMBs.^12,14,15,45,87^ In fact, high values of dielectric constants, Born effective charges, and low thermal conductivity have been discussed for many PVs, especially for BaTiO_3_.^5^ Note that metallization (closing of bandgap) is predicted when EDMBs are formed in the *ab* plane of the *P*4*/nmm* phase and that the values of dielectric constant and Born effective charges along the *c*-axis of the *P*4*/nmm* phase do not show high values near the phase transition unlike what happens to the components in the *ab* plane. It must be noted that the metallic or quasi-metallic behavior of the tetragonal PV phase of CsIO_3_ is similar to that of the tetragonal (*P*4/*mmm*) PV phase of La_0.5_Ba_0.5_CoO_3_ ^88^ and the ordered tetragonal (*P*4/*mmm*) PV phase of LaBaCo_2_O_6_ which also show CoO_5_ units^89^ (see also chapter 4 in ref 3).

**Figure 8:**
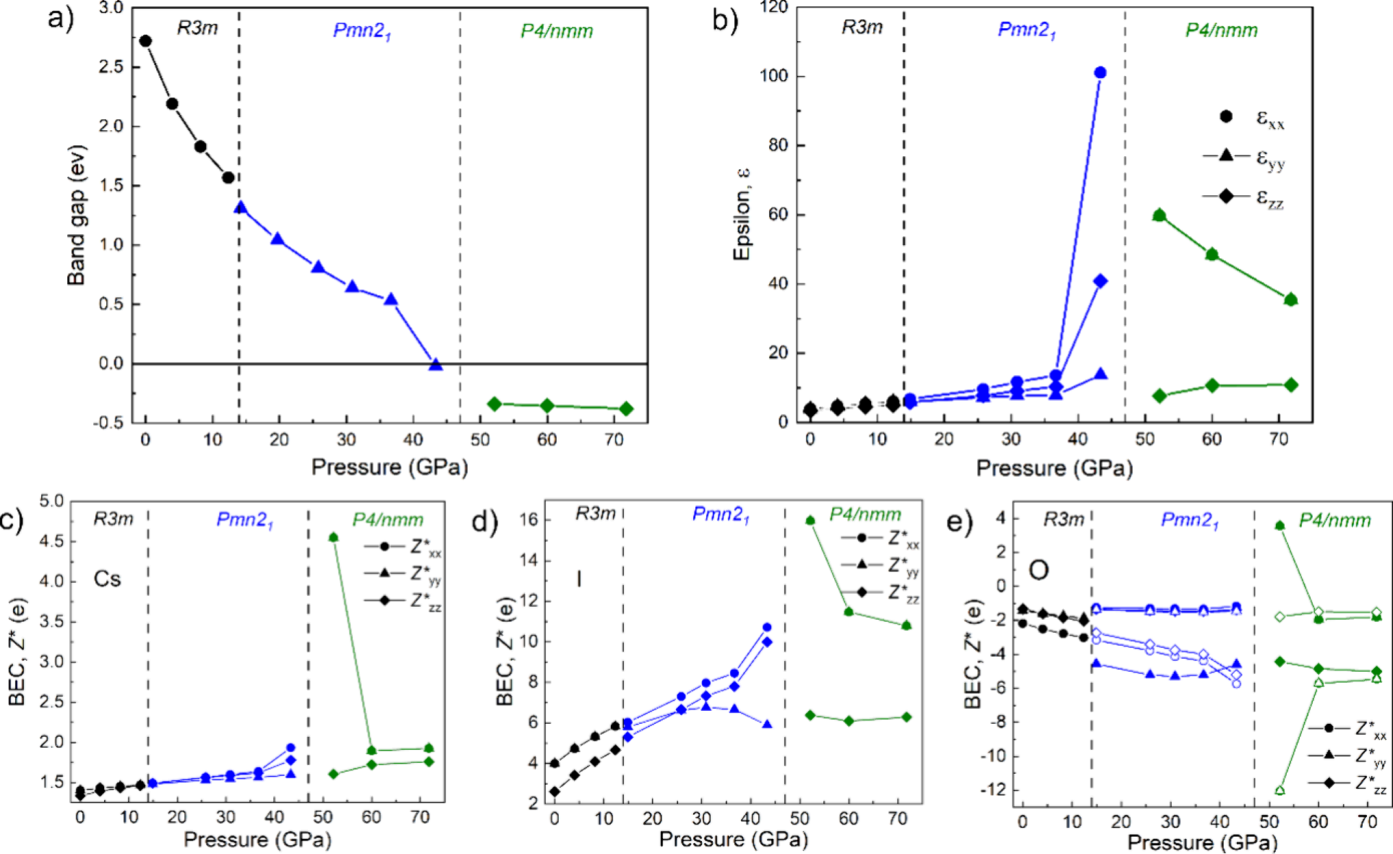
Pressure dependence of the bandgap (a), dielectric constant (b), and Born effective charges of Cs (c), I (d), and O (e) atoms in the different phases of CsIO_3_. A metallization is predicted at pressures close to that of the *Pmn*2_1_-*P*4/*nmm* phase transition; i.e., upon formation of EDMBs. Similarly, an extraordinary increase of the dielectric constant and Born effective charges occur near the phase transition, as it occurs in pnictogens, chalcogens, and phase change materials when EDMBs are formed.

As a final comment regarding the comparison of perovskite CsIO_3_ and phase change materials, it is interesting to note that the pressure-induced hypercoordination of I atoms in CsIO_3_ is similar to that found in SnSe. In the orthorhombic *Pnma* phase (SG No. 62) of SnSe at RP, Sn atoms are 3-fold coordinated as I in the *R*3*m* of CsIO_3_. Around 10 GPa, SnSe undergoes a phase transition toward the orthorhombic *Cmcm* (or *Bbmm*) phase (SG No. 63)^90,91^ and the *Cmcm* phase is characterized by square pyramidal SnSe_5_ units similar to those IO_5+1_ of Figure 3b. An increase of the short covalent Sn–Se bonds in the *ab* plane occurs in the *Pnma* phase, while the covalent Sn–Se bond along the *c* axis compresses in a normal way.^90^ A softening of the high-wavenumber vibrational modes related to Sn–Se bonds in the *ab* plane is also observed.^91^ A change is observed once the *Cmcm* phase is reached. All Sn–Se bonds compress in this phase and all vibrational modes show a positive pressure coefficient. This change has been ascribed to the formation of EDMBs in the square plane of the SnSe_5_ unit, while a covalent Sn–Se bond (the shortest bond) still persists along the *c-*axis of the *Cmcm* phase.^15^

### 4.5 Trends in *A*IO_3_ Compounds under Compression

It has been already suggested that phase transitions in the distorted PV structures of *A*IO_3_ (*A* = K, Rb, Cs, Tl) compounds would occur at smaller pressures under non-hydrostatic conditions than under hydrostatic conditions, as indeed found in KIO_3_.^37^ In this context, a change in the pressure coefficients of the high-wavenumber phonons was already observed in KIO_3_ above 14 GPa^37^ despite our calculations for this compound in the *R*3*m* phase do not support a PIS under hydrostatic conditions until ca. 35 GPa (see Figure 9). Note that the I–O bond distances in the simulated *R*3*m* phases of *A*IO_3_ (*A* = K, Rb, Cs, Tl) compounds show a PIS in all of them (see Figure 9).

**Figure 9:**
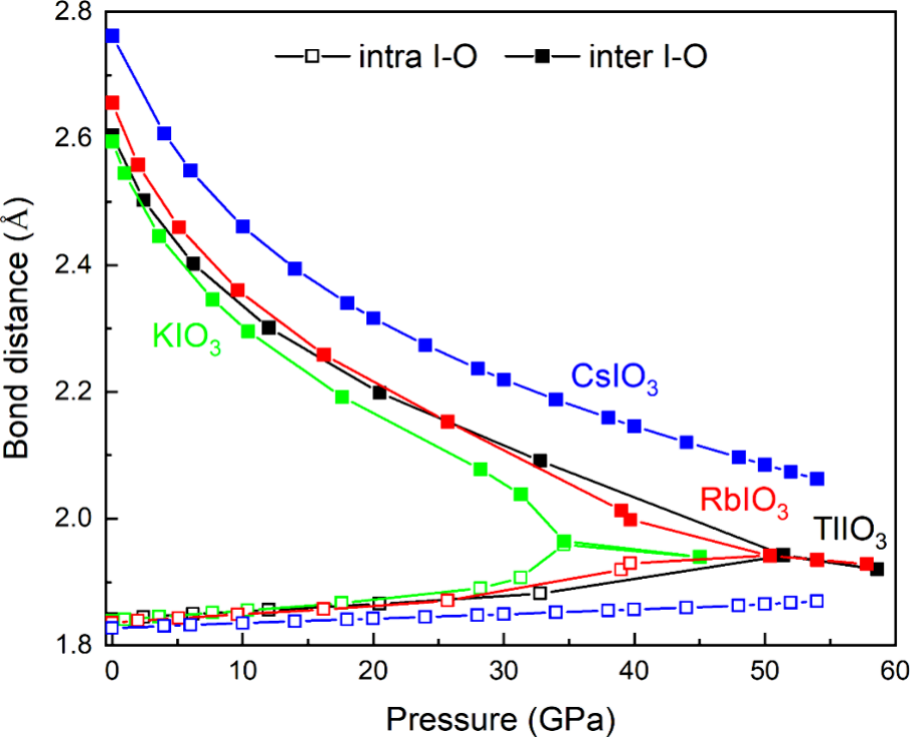
Pressure dependence of the simulated short (intramolecular) I–O and long (intermolecular) I···O bond distances in the simulated *R*3*m* phases of several *A*IO_3_ (*A* = K, Rb, Cs, and Tl) compounds.

Our results confirm that *A*IO_3_ (*A* = K, Rb, Cs, Tl, NH_4_) compounds undergo a PIS and tend to form the cubic PV structure at HP. The complete PIS does not occur in CsIO_3_, which remains with a tetragonal *P*4*/nmm* structure even up to 70 GPa. However, the complete PIS happens already at 60 GPa for RbIO_3_, which shows a quasi-cubic rhombohedral *R*-3*m* phase (Figure S8). Therefore, we conclude that RbIO_3_ and TlIO_3_ are excellent candidates to verify experimentally the formation of EDMBs during the formation of the PV structure at HP. It can be observed that there is a correlation between the PIS pressure of *A*IO_3_ compounds and the ionic size radius, *r*, of the *A* cation (*r*_K_ < *r*_Rb_ ≈ *r*_Tl_ < *r*_Cs_).^92^ This result supports our idea that the A cation acts as a stabilizing unit of the PV structure and that the large ionic size of Cs is behind the lack of full PIS of CsIO_3_ as previously commented.

It is important to notice that the PIS is associated with a pressure-induced polymerization (PIP) process of the IO_3_ units. The PIP process of the IO_3_ units (monomers) leads to the formation of concatenated IO_5+1_ units along two spatial directions (in CsIO_3_) or IO_6_ units along three spatial directions (in other *A*IO_3_ compounds). Consequently, the polymerization of the IO_3_ units results in the formation of EDMBs at HP in both the tetragonal and (quasi-) cubic phases of *A*IO_3_ compounds.

A simple way of understanding the PIP process of IO_3_ units to yield IO_6_ units in *A*IO_3_ compounds at HP is shown in Figure 10. To avoid complications related to the change of space group of the crystalline structure and bond angles, etc., we could assume that *A*IO_3_ compounds at RP have a cubic unit cell of lattice parameter *a* (*a*_0_ at RP) in which isolated IO_3_ units (monomers) exhibit three short covalent I–O bonds (of bond length *d*) directed along the *a*, *b*, and *c*-axes, respectively. This means that all O–I–O angles are 90°. Let us also assume for simplicity that the IO_3_ units are characterized by short primary covalent I–O bonds with bond length *d* = *a*_0_/3 at RP. This configuration means that each I atom shows also three long secondary noncovalent I–O interactions (along each of the three axes) with bond length *d*’ = 2*a*_0_/3 at RP. This situation is described in Figure 10a, where the two types of I–O bonds lead to asymmetric IO_6_ octahedra. Now let us imagine that at HP this cubic cell compresses (the lattice parameter *a* decreases) and we observe that *d* enlarges while *d*’ compresses until both I–O bond lengths are *d* = *d*’ = *a*/2 at a certain pressure (the phase transition pressure, *P*_T_). This situation is described in Figure 10b, where there is only one type of bond resulting in symmetric IO_6_ octahedra characteristic of the PV structure obtained after the PIP process of IO_3_ units. To understand the PIP process of IO_3_ units from the point of view of the symmetrization of the two types of I–O bonds, the behavior of the *d* and *d*’ values (in relation to *a*) with increasing pressure is shown in Figure 10c. In this simplified way, we can understand the PIP process that transforms all isolated IO_3_ units (belonging to asymmetric IO_6_ units) into concatenated IO_3_ units (belonging to symmetric IO_6_ units). This PIP process explains how the cubic PV structure is obtained in *A*IO_3_ compounds under compression.

**Figure 10:**
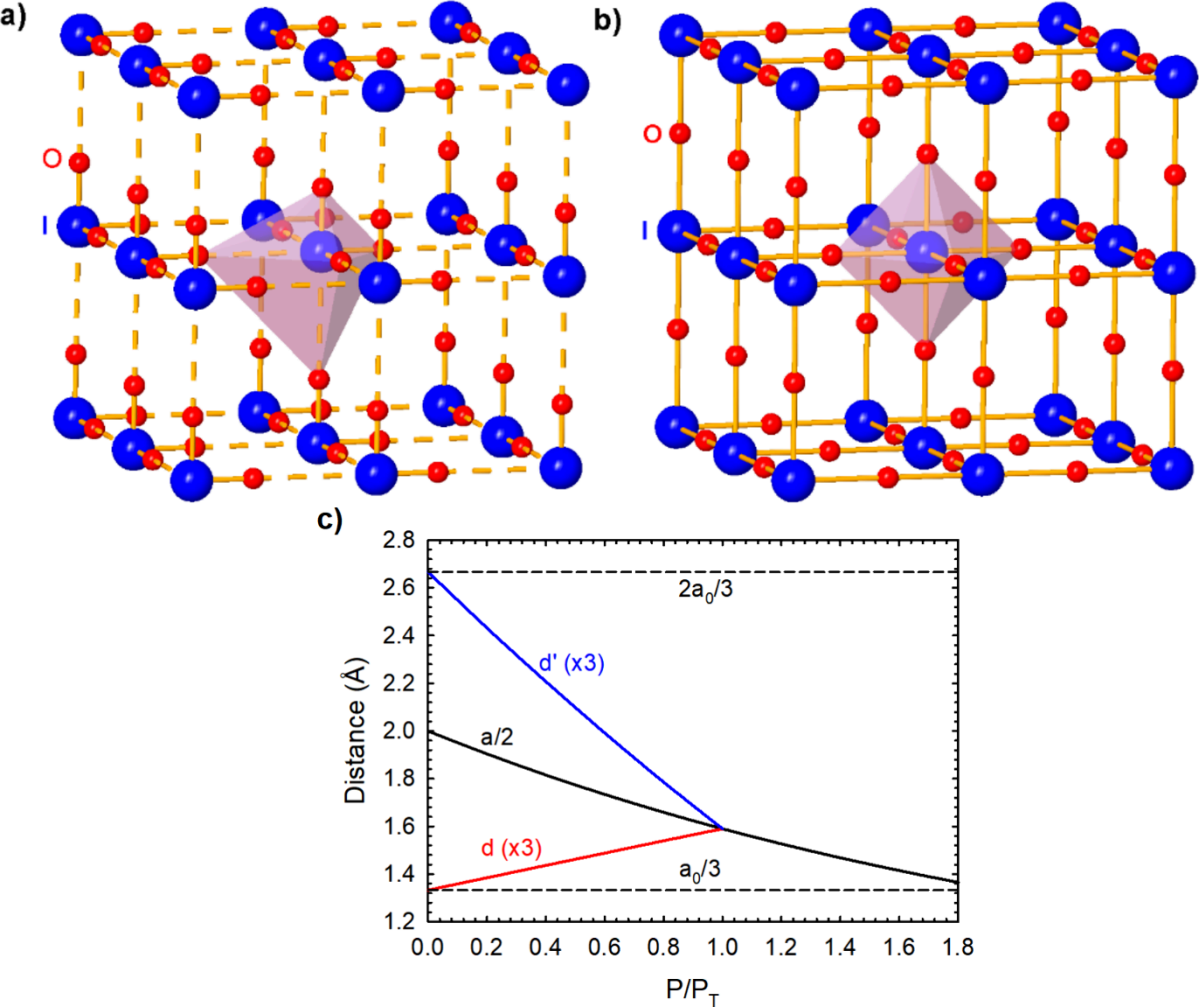
Simplified scheme of the pressure-induced polymerization (PIP) process of IO_3_ units in *A*IO_3_ compounds leading to the IO_6_ units of the cubic perovskite (PV) structure. Big blue atoms represent I atoms and small red atoms represent O atoms. To focus on the PIP process of IO_3_ units, *A* atoms (inside the cubes) are not shown. (a) Room pressure (RP): Cubic structure of a strongly distorted PV with highly asymmetric IO_6_ units due to the existence of three short (solid) and three long (dashed) I–O bonds characteristic of isolated IO_3_ units. (b) High pressure: Cubic PV structure showing symmetric IO_6_ units with six equal I–O bond lengths. (c) Pressure dependence of the *a* lattice parameter and the three short (*d*) and three long (*d*’) I–O bond distances (in terms of *a*). The value of *a* at RP, *a*_0_, has been taken as 4 Å, and the *d* and *d*’ values at RP have been taken as *a*_0_/3 and 2*a*_0_/3, respectively. *P*_T_ indicates the phase transition pressure at which the cubic PV structure is reached and the PIP process ends. Note the different compression of bond lengths before and after *P*_T_; i.e., before and after EDMBs in IO_6_ units are formed.

In summary, all *A*IO_3_ (*A* = K, Rb, Cs, Tl, NH_4_) compounds crystallize at RP in strongly distorted perovskite structures characterized by IO_3_ units with delocalized resonant (covalent-like) I–O bonds, resulting from the strong distortion of IO_6_ polyhedra due to the strength of the iodine LEP. As pressure increases, the LEP becomes progressively less active, as found in many studies.^17–23^ Our experiments and calculations show that *A*IO_3_ (*A* = K, Rb, Cs, Tl, NH_4_) compounds tend to symmetrize under compression, forming cubic PVs or slightly distorted PVs. These structures have regular or slightly distorted IO_6_ units with EDMBs; i.e., 2c–1e I–O bonds that come from the PIP process of the IO_3_ units leading to IO_5+1_ and IO_6_ units via concatenated linear or quasi-linear 3c–2e bonds in 2D and 3D, respectively. Therefore, the PV structure to be formed in *A*IO_3_ compounds at HP can be considered as a PIP process of the IO_3_ units to form IO_6_ units. That polymerization involves a change in chemical bonding from delocalized resonant (covalent-like) I–O bonds in the IO_3_ units toward 3D I–O EDMBs in the IO_6_ units. In CsIO_3_, the tetragonal perovskite is found above 45 GPa with well-defined 2D I–O EDMBs in the *ab-*plane. Along the *c*-axis a weakened covalent bond and strengthened noncovalent bond still persist in this tetragonal phase and the cubic PV structure is not observed even up to 70 GPa likely due to steric repulsions related to the size of Cs atom. On the contrary, full PIS leading to a cubic PV structure is expected in *A*IO_3_ compounds with *A* ^+^ cations showing a smaller ionic radius than Cs.

## 5 Conclusions

Through a joint HP experimental and theoretical study of the distorted perovskite CsIO_3_, we have shown that the rhombohedral (*R*3*m*) phase at room conditions undergoes two pressure-induced phase transitions up to 70 GPa. The first transition occurs around 14 GPa, yielding an orthorhombic phase (*Pmn*2_1_), while the second one, leads to a tetragonal perovskite phase (*P*4/*nmm*). These transformations precede a potential transition to the cubic (*Pm*-3*m*) perovskite structure that could happen at pressures well above 70 GPa.

Our study reveals a gradual transformation of the initial IO_3_ units of CsIO_3_ into slightly distorted IO_5+1_ units as the tetragonal (quasi-cubic) phase is approached; i.e., a pressure-induced polymerization of the IO_3_ units is observed that leads to I hypercoordination. Concomitantly, the I–O bonds change from the short primary delocalized resonant (covalent-like) bonds in IO_3_ units toward electron-deficient multicenter bonds (EDMBs) as the polymerization proceeds under compression. In other words, I–O bonds become 2c–1e EDMBs in IO_5+1_ units of the tetragonal PV phase of CsIO_3_ due to the progressive polymerization of IO_3_ units. The equalization of bond distances and the hardening of the originally soft high-wavenumber Raman-active modes, once the bond distances equalize, give support to the formation of EDMBs, in agreement with the recently proposed theory of multicenter bonding.^14,15^

The results of this work can be extrapolated to the whole family of *A*IO_3_ (*A* = K, Rb, Cs, Tl, NH_4_) compounds, where EDMBs will be developed at high pressure as the cubic perovskite structure is approached upon polymerization of IO_3_ units, being RbIO_3_ and TlIO_3_ ideal candidates to observe the EDMBs in the three directions of space once the complete pressure-induced symmetrization of the perovskite structure occurs due to the complete pressure-induced polymerization process of the IO_3_ units.

The results of this work, together with previous findings indicating that lead halide perovskites feature electron-deficient metavalent bonds^11^ (here understood as EDMBs^14,15^ ), suggest that the cubic perovskite *ABX*_3_ structure of main-group elements and their slightly distorted variants is fundamentally governed by a combination of ionic bonds (between the *A* cation and *X* anions) and EDMBs (between the *B* cation and *X* anions). It has been considered that oxide perovskites, like BaTiO_3_, do not show this type of bonding.^11^ This could lead to think that oxide perovskites in general do not show this type of bonding. In contrast, we have shown here that this unconventional bonding is present in *A*IO_3_ perovskites. This unconventional bonding interplay may account for many of the extraordinary properties found in main-group perovskites, which cannot be explained within the framework of classic (covalent, ionic, and metallic) bonding. We hope that our findings will stimulate more studies on the chemical bonding in other perovskites (w/o main-group elements) to evaluate the extent to which the concept of EDMB can be applied in perovskites.

## Supplementary Material



## Notes

Details of the structural, vibrational, and chemical properties of CsIO_3_ and RbIO_3_ under compression; equations of state of the different phases of CsIO_3_; pressure dependence of experimental and theoretical atomic parameters in the orthorhombic phase; and tables with structural, vibrational, and chemical data of the different phases of CsIO_3_ and RbIO_3_ (PDF)

## References

[1] Properties and Applications of Perovskite-type Oxides; CRC Press: New York, 1993.

[2] Goodenough J. B. (2004). Electronic
and ionic transport properties and other physical aspects of perovskites. Rep. Prog. Phys..

[3] Perovskites: structure, properties, and uses; Nova Science Publications: New York, 2010.

[4] Fedorov, V. ; Perovskites, in Ceramics Science and Technology Vol. 2: Properties; Weinheim: Wiley-VCH, 2010.

[5] Ghosez Ph., Cockayne E., Waghmare U. V. (1999). Lattice dynamics of
BaTiO_3_, PbTiO_3_, and PbZrO_3_: A comparative
first-principles study. Phys. Rev. B.

[6] Raveau B. (1986). Numerous structures
of oxides can be built up from the ReO_3_-type framework. Proc. Indian Acad. Sci. Part A.

[7] Evans H. A., Wu Y., Seshadri R. (2020). Perovskite-related ReO_3_-type
structures. Nat. Rev. Mater..

[8] Du M., Huang H., Zhang Z. (2024). High-Temperature Superconductivity
in Perovskite Hydride Below 10 GPa. Adv. Sci..

[9] Hahn U., Weber W. (1996). Electronic structure and chemical-bonding
mechanism of Cu_3_N, Cui_3_NPd, and related Cu­(I)
compounds. Phys. Rev. B.

[10] Yu W., Zhao J. G., Jin C. Q. (2005). Simultaneous
softening of Cu_3_N phonon modes along the *T*
_2_ line
under pressure: A first-principles calculation. Phys. Rev. B.

[11] Wuttig M., Schön C.-F., Schumacher M. (2022). Halide perovskites:
Advanced photovoltaic materials empowered by a unique bonding mechanism. Adv. Funct. Mater..

[12] Wuttig M., Deringer V. L., Gonze X. (2018). Incipient metals: functional
materials with a unique bonding mechanism. Adv.
Mater..

[13] Müller P. C., Elliott S. R., Dronskowski R. (2024). Chemical bonding in
phase-change chalcogenides. J. Phys.: Condens.
Matter.

[14] Osman H. H., Otero-de-la-Roza A., Munoz A., Manjón F. J. (2024). Electron-deficient
multicenter bonding in pnictogens and chalcogens: Mechanism of formation. J. Mater. Chem. C.

[15] Osman H. H., Rodríguez-Hernández P., Muñoz A., Manjón F. J. (2025). A Unified Theory of Electron-Rich and Electron-Deficient
Multicenter Bonds in Molecules and Solids: A Change of Paradigms. J. Mater. Chem. C.

[16] Bader R. F. W. (1985). Atoms
in molecules. Acc. Chem. Res..

[17] Bandiello, E. ; Lobato, A. ; Izquierdo, F. From Polyanions to Infinite Chains: Chemical Bonding Evolution in AX_3_ Polyhalides under Pressure. ChemRxiv 2025 10.26434/chemrxiv-2025-gdx2s

[18] Pereira A. L. J., Gomis O., Sans J. A. (2014). Pressure effects on
the vibrational properties of α-Bi_2_O_3_:
an experimental and theoretical study. J. Phys.:
Condens. Matter.

[19] Pereira A. L. J., Sans J. A., Vilaplana R. (2014). Isostructural Second-Order
Phase Transition of β-Bi_2_O_3_ at High Pressures:
An Experimental and Theoretical Study. J. Phys.
Chem. C.

[20] Pereira A.
L. J., Gomis O., Sans J. A. (2016). β–Bi_2_O_3_ under compression:
Optical and elastic properties
and electron density topology análisis. Phys. Rev. B.

[21] Ibañez J., Sans J. A., Popescu C. (2016). Structural, Vibrational,
and Electronic Study of Sb_2_S_3_ at High Pressure. J. Phys. Chem. C.

[22] Cuenca-Gotor V. P., Sans J. A., Gomis O. (2020). Orpiment
under compression:
metavalent bonding at high pressure. Phys. Chem.
Chem. Phys..

[23] Sans J. A., Manjón F. J., Pereira A. L. J. (2021). Unveiling the role of
the lone electron pair in sesquioxides at high pressure: Compressibility
of β-Sb_2_O_3_. Dalton
Transactions.

[24] Zhang X. W., Abdalla L. B., Liu Q. H. (2017). The Enabling Electronic
Motif for Topological Insulation in ABO_3_ Perovskites. Adv. Funct. Mater..

[25] Herlach F. (1961). Kernquadrupolresonanzen,
Phasenumwandlungen und Ferroelektrizität der Alkalijodate. Helv. Phys. Acta.

[26] Bergman J. G., Boyd G. D., Ashikin A. (1969). New nonlinear optical
materials: metal oxides with nonbonded electrons. J. Appl. Phys..

[27] Xin Y., Mengkai L., Shaojun Z. (1992). Nonlinear optical properties
of perfectly polarized KIO_3_ single crystal. Chin. Phys. Lett..

[28] Zhang M., Hu C., Abudouwufu T. (2018). Functional materials design via structural
regulation originated from ions introduction: A study case in cesium
iodate system. Chem. Mater..

[29] Crane G. R. (1972). The relation
of physical properties to the symmetry of potassium iodate. J. Appl. Crystallogr..

[30] Wu Q., Liu H., Jiang F. (2016). RbIO_3_ and
RbIO_2_F_2_: Two Promising Nonlinear Optical Materials
in Mid-IR Region
and Influence of Partially Replacing Oxygen with Fluorine for Improving
Laser Damage Threshold. Chem. Mater..

[31] Hamid S. A. (1973). Symmetrie
von KIO_3_ und die Struktur der Zimmertemperaturphase. Z. Kristallogr..

[32] Kalinin V. R., Ilyvkhin V. V., Belov N. V. (1978). On Crystal structure
of triclinic
modification of potassium iodate. Dokl. Akad.
Nauk SSSR.

[33] Lucas B. W. (1984). Structure
(neutron) of room-temperature phase III potassium iodate, KIO_3_. Acta Crystallogr. C.

[34] Huang J., Guo F., Guo Z. (2022). NH_4_IO_2_F_2_ and
(NH_4_)_3_(IO_2_F_2_)_3_·H_2_O: A Series of Ammonium-Containing Fluoroiodates
with Wide Band Gaps. Inorg. Chem..

[35] Alcock N. W. (1972). The crystal
structure of α-rubidium iodate. Acta Cryst.
B.

[36] Bergman J. G., Wood J. S. (1987). Structure of thallium
(I) iodate. Acta Cryst. C.

[37] Bayarjargal L., Wiehl L., Friedrich A. (2012). Phase transitions in
KIO_3_. J. Phys.: Condens. Matter.

[38] Liang A., Popescu C., Manjón F. J. (2021). Pressure-Driven Symmetry-Preserving
Phase Transitions in Co­(IO_3_)_2_. J. Phys. Chem. C.

[39] Liang A., Popescu C., Manjón F. J. (2021). Structural and vibrational
study of combining high-pressure experiments and density-functional
theory. Phys. Rev. B.

[40] Liang A., Rahman S., Rodríguez-Hernández P. (2020). High-Pressure Raman Study of Fe­(IO_3_)_3_: Soft-Mode
Behavior Driven by Coordination Changes of Iodine Atoms. J. Phys. Chem. C.

[41] Errandonea D., Osman H. H. H., Turnbull R. (2024). Pressure-induced hypercoordination
of iodine and dimerization of I_2_O_6_H in strontium
di-iodate hydrogen-iodate (Sr­(IO_3_)­2HIO_3_). Mater. Today Adv..

[42] Kim M., Yoo C.-S. (2016). Phase transitions in I_2_O_5_ at
high pressures: Raman and X-ray diffraction studies. Chem. Phys. Lett..

[43] Sharma B. B., Ghosh P. S., Mishra A. K. (2021). Hyper-coordinated
iodine
in HIO_3_ under pressure. Vibrat. Spectr..

[44] Liang A., Turnbull R., Errandonea D. (2023). A review on
the advancements in the
characterization of the high-pressure properties of iodates. Prog. Mater. Sci..

[45] Manjón F. J., Osman H. H., Savastano M. (2024). Electron-Deficient Multicenter
Bonding in Phase Change Materials: A Chance for Reconciliation. Materials.

[46] Klotz S., Chervin J.-C., Munsch P. (2009). Hydrostatic limits of
11 pressure transmitting media. J. Phys. D:
Appl. Phys..

[47] Errandonea D., Muñoz A., Gonzalez-Platas J. (2014). Comment on High-pressure x-ray diffraction
study of YBO_3_/Eu^3+^, GdBO_3_, and EuBO_3_: Pressure-induced amorphization in GdBO_3_. J. Appl. Phys..

[48] Fauth F., Peral I., Popescu C. (2013). The new material science
powder diffraction beamline at ALBA synchrotron. Powder Diffraction.

[49] Dewaele A., Torrent M., Loubeyre P. (2008). Compression curves of
transition metals in the Mbar range: Experiments and projector augmented-wave
calculations. Phys. Rev. B.

[50] Prescher C., Prakapenka V. B. (2015). DIOPTAS:
a program for reduction of two-dimensional
X-ray diffraction data and data exploration. High Press. Res..

[51] Rodríguez-Carvajal J. (1993). Recent advances
in magnetic structure determination by neutron powder diffraction. Phys. B Phys. Condens Matter.

[52] Syassen K. (2008). Ruby under
pressure. High Press. Res..

[53] Kresse G., Furthmuller J. (1996). Efficiency
of ab-initio total energy calculations for
metals and semiconductors using a plane-wave basis set. Comput. Mater. Sci..

[54] Kresse G., Furthmüller J. (1996). Efficient iterative schemes for ab
initio total-energy
calculations using a plane-wave basis set. Phys.
Rev. B.

[55] Kresse G., Joubert D. (1999). From ultrasoft pseudopotentials to the projector augmented-wave
method. Phys. Rev. B.

[56] Kresse G., Furthmuller J., Hafner J. (1994). Theory of the crystal structures
of selenium and tellurium: the effect of generalized-gradient corrections
to the local-density approximation. Phys. Rev.
B.

[57] Blochl P. E. (1994). Projector
augmented-wave method. Phys. Rev. B.

[58] Perdew J. P., Ruzsinszky A., Csonka G. I. (2008). Restoring the density-gradient
expansion for exchange in solids and surfaces. Phys. Rev. Lett..

[59] Monkhorst H. J. (1976). Special
points for Brillouin-zone integrations. Phys.
Rev. B.

[60] Togo A., Oba F., Tanaka I. (2008). First-principles
calculations of the ferroelastic transition
between rutile-type and -type at high pressures. Phys. Rev. B.

[61] Giannozzi P., Andreussi O., Brumme T. (2017). Advanced capabilities
for materials modelling with Quantum ESPRESSO. J. Phys.: Condens. Matter..

[62] Mostofi A. A., Yates J. R., Lee Y.-S. (2008). wannier90: A tool for
obtaining maximally-localised Wannier function. Comput. Phys. Commun..

[63] Mostofi A. A., Yates J. R., Lee Y.-S. (2014). An
updated version of
wannier90: A tool for obtaining maximally-localised Wannier functions. Comput. Phys. Commun..

[64] Otero-de-la-Roza A., Johnson E. R., Luaña V. (2014). Critic2: A
program for real-space
analysis of quantum chemical interactions in solids. Comput. Phys. Commun..

[65] Dal
Corso A. (2014). Pseudopotentials periodic table: From H to Pu. Comput. Mater. Sci..

[66] Otero-de-la-Roza A., Martín Pendás Á., Johnson E. R. (2018). Quantitative electron
delocalization in solids from maximally localized Wannier functions. J. Chem. Theory Comput..

[67] Momma K., Izumi F. (2011). VESTA 3 for three-dimensional visualization
of crystal, volumetric
and morphology data. J. Appl. Crystallogr..

[68] Wuttig M., Schön C.-F., Kim D. (2023). Metavalent or hypervalent
bonding: is there a chance for reconciliation?. Adv. Sci..

[69] Mukhopadhyay S., Sun J. F., Subedi A. (2016). Competing covalent and
ionic bonding in Ge-Sb-Te phase change materials. Sci. Rep..

[70] Lee T. H., Elliot S. R. (2017). The Relation between
Chemical Bonding and Ultrafast
Crystal Growth. Adv. Mater..

[71] Goldschmidt V. M., Barth T., Lunde G. V. V. (1928). Untersuchungen ueber die Kristallstruktur
von Sesquioxyden und Verbindungen ABO_3_. Norwegian Acad. Sci. Lett..

[72] All phases described in this work for CsIO_3_ (*R*3*m*, *Pmn*2_1_, and *P*4/*nmm*) show the same enthalpy vs pressure within the uncertainty of our calculations.

[73] Blundell, S. J. ; Blundell, K. M. Concepts in Thermal Physics; Oxford University Press, 2008.

[74] Mori S., Hatayama S., Shuang Y. (2020). Reversible displacive
transformation in MnTe polymorphic semiconductor. Nat. Commun..

[75] Erkişi A., Gökoğlu G., Sürücü G. (2016). First-principles investigation
of LaGaO_3_ and LaInO_3_ lanthanum perovskite oxides. Philos.
Mag..

[76] Salje E. (1976). Symmetry and
lattice dynamics of oxides with perovskite-like structures. Acta Cryst. A.

[77] Kroumova E., Aroyo M. L., Perez-Mato (2003). Bilbao Crystallographic
Server: Useful Databases
and Tools for Phase-Transition Studies. Phase
Transitions.

[78] Pyykkö P., Atsumi M. (2009). Molecular Single-Bond Covalent Radii for Elements 1–118. Chemistry: A European Journal.

[79] Pyykkö P., Atsumi M. (2009). Molecular Double-Bond
Covalent Radii for Elements Li–E112. Chemistry: A European Journal.

[80] Fjellvag H., Kjekshus A. (1994). The Crystal Structure of I_2_O_4_ and its Relations to Other Iodine-Oxygen-Containing Compounds. Acta Chem. Scand..

[81] Kiprof P. (2005). The nature
of iodine oxygen bonds in hypervalent 10-I-3 iodine compounds. Arkivoc.

[82] Berski S., Latajka Z., Gordon A. J. (2011). Oxygen bound iodine
(O–I):
The Electron Localization Function (ELF) study on bonding in cis-
and trans-IONO. Chem. Phys. Lett..

[83] Teichtmeister T. A., Johrendt D., Bernhart A. H., Heymann G., Huppertz H. (2024). A Comparative Study
of a High-Pressure Polymorph of I_2_O_5_ and its
Ambient-Pressure Modification. Chem.–Eur.
J..

[84] Kraft T., Jansen M. (1995). Synthesis and Crystal Structure of
Diiodine (V/VII)
Hexaoxide: An Intermediate between a Molecular and a Polymer Solid. J. Am. Chem. Soc..

[85] Straus D. B., Mitchell Warden H. E., Cava R. J. (2021). s–p Mixing in Stereochemically
Active Lone Pairs Drives the Formation of 1D Chains of Lead Bromide
Square Pyramids. Inorg. Chem..

[86] Tang B., Hu Y. J., Dong H. X. (2019). An All-Inorganic Perovskite-Phase
Rubidium Lead Bromide Nanolaser. Angew. Chem.,
Int. Ed..

[87] Lee S. Y., Esfarjani K., Luo T. F. (2014). Resonant bonding leads
to low lattice thermal conductivity. Nat. Commun..

[88] Fauth F., Suard E., Caignaert V. (2001). Intermediate
spin state of Co^3+^ and Co^4+^ ions in La_0.5_Ba_0.5_CoO_3_ evidenced by Jahn-Teller distortions. Phys. Rev. B.

[89] Rautama E. L., Boullay P., Kundu A. K. (2008). Cationic
Ordering and
Microstructural Effects in the Ferromagnetic Perovskite La_0.5_Ba_0.5_CoO_3_: Impact upon Magnetotransport Properties. Chem. Mater..

[90] Loa I., Husband R. J., Downie R. A. (2015). Structural changes in
thermoelectric SnSe at high pressures. J. Phys.:
Condens. Matter.

[91] Efthimiopoulos I., Berg M., Bande A. (2019). Effects
of temperature
and pressure on the optical and vibrational properties of thermoelectric
SnSe. Phys. Chem. Chem. Phys..

[92] Shannon R. D. (1976). Revised
Effective Ionic Radii and Systematic Studies of Interatomic Distances
in Halides and Chalcogenides. Acta Cryst. A.

