# Supplementary material for: Unconventional Electron-Deficient Multicenter Bonds in AIO3 Perovskites
Source: Chem Mater. 2025 May 30;37(11):4187–202. doi: 10.1021/acs.chemmater.5c00877 (PMC12160587; doi:10.1021/acs.chemmater.5c00877)
Supplement: Supplementary file 1 [file cm5c00877_si_001.pdf]

Supporting Information

of

Unconventional electron-deficient multicenter bonds in AIO_3 perovskites

Hussien H. Osman[1,2,3], José Luis Rodrigo-Ramón[2], Shafi Ullah[1], Enrico Bandiello[1], Daniel Errandonea[2], Óscar Gomis[4], Tania García-Sánchez[1], Pablo Botella[2], Robert Oliva[5], Plácida Rodríguez-Hernández[6], Alfonso Muñoz[6], Catalin Popescu[7], Frederico G. Alabarse[8], and Francisco Javier Manjón[1*]

[1] Instituto de Diseño para la Fabricación y Producción Automatizada, MALTA Consolider Team, Universitat Politècnica de València, 46022, València, Spain

[2] Instituto de Ciencia de los Materiales de la Universitat de València, MALTA Consolider Team, Universitat de València, 46100, Valencia, Spain

[3] Chemistry Department, Faculty of Science, Helwan University, 11795, Cairo, Egypt

[4] Centro de Tecnologías Físicas, MALTA Consolider Team, Universitat Politècnica de València, 46022, València, Spain

[5] Geosciences Barcelona (GEO3BCN), MALTA Consolider Team, CSIC, Lluís Solé i Sabarís s/n, 08028 Barcelona, Catalonia, Spain

[6] Departamento de Física, MALTA Consolider Team, Universidad de La Laguna, 38205, La Laguna, Tenerife, Spain

[7] ALBA-CELLS, MALTA Consolider Team, 08290 Cerdanyola del Valles (Barcelona), Catalonia, Spain

[8] Elettra Sincrotrone Trieste, S.S. 14 - Km 163,5 in AREA Science Park, 34149, Basovizza, Trieste, Italy

*Corresponding author(s). E-mail(s): fjmanjon@fis.upv.es

1. Structural properties

Two powder angle-resolved X-ray diffraction experiments were performed at high pressure conditions (at ALBA and Elettra). In ALBA experiments, the obtained unit-cell parameters of $CsIO_3$ at room pressure were: a = 6.606(4) Å and c = 8.102(5) Å. In the Elettra experiments, the obtained unit-cell parameters of the rhombohedral phase (SG $R3m$, No. 160, Z=1) at room pressure are a = 6.600(4) Å and c = 8.093(5) Å. Both sets of parameters agree with those earlier reported: a = 6.6051 Å and c = 8.0870 Å **[1]**.

The X-ray diffraction patterns measured in the experiment performed at ALBA (**Figure S1**) are consistent with the rhombohedral structure up to 12.6(1) GPa. By comparing the patterns at this pressure and at room pressure, the splitting of several peaks that are degenerated at room pressure, like (012)/(110) and (003)/(021), can be observed. In particular, the rhombohedral structure fails to explain well the shape of the strongest peak and the shift to higher angles of the peak at 11.4°. We found that the X-ray diffraction pattern measured at 14.4(1) GPa can be better explained by an orthorhombic $Pmn2_1$ (No. 31) structure. Rietveld refinements with both structures show that

the orthorhombic model fits better the X-ray diffraction data at 14.4(1) GPa. A comparison of both Rietveld refinements is shown in **Figure S3**. This structure can reasonably explain the X-ray diffraction patterns collected up to the highest pressure (see the pattern measured at 40.6(1) GPa in **Figure S1**). Upon decompression, we collected a few X-ray diffraction patterns. We observed the orthorhombic structure at 28.1(1) and 18.6(1) GPa and the rhombohedral structure at 8.05(5) and 1.05(5) GPa, thus indicating the reversibility of the pressure-induced trigonal-orthorhombic phase transition. The X-ray diffraction pattern measured at the lowest pressure and the Rietveld refinement performed assuming the rhombohedral structure are shown at the top of **Figure S1**.

In the experiments performed at Elettra we found similar results. The trigonal phase was found up to 13.7(1) GPa and the orthorhombic phase was found at the next compression step at 15.9(1) GPa. The subtle changes of X-ray diffraction indicating the transition can be seen in **Figure S2** by comparing the X-ray diffraction patterns measured at 13.7(1) GPa and 19.4(1) GPa. Combining the results from both experiments, we can state that the onset of the transition is at 14.4(1) GPa. In the Elettra experiment, the orthorhombic phase was observed up to the highest pressure covered by the experiment, 34.9(1) GPa. Under decompression, the high-pressure phase was observed up to 14.7(1) GPa, and at the next decompression step, we found the trigonal structure at 10.8(1) GPa.

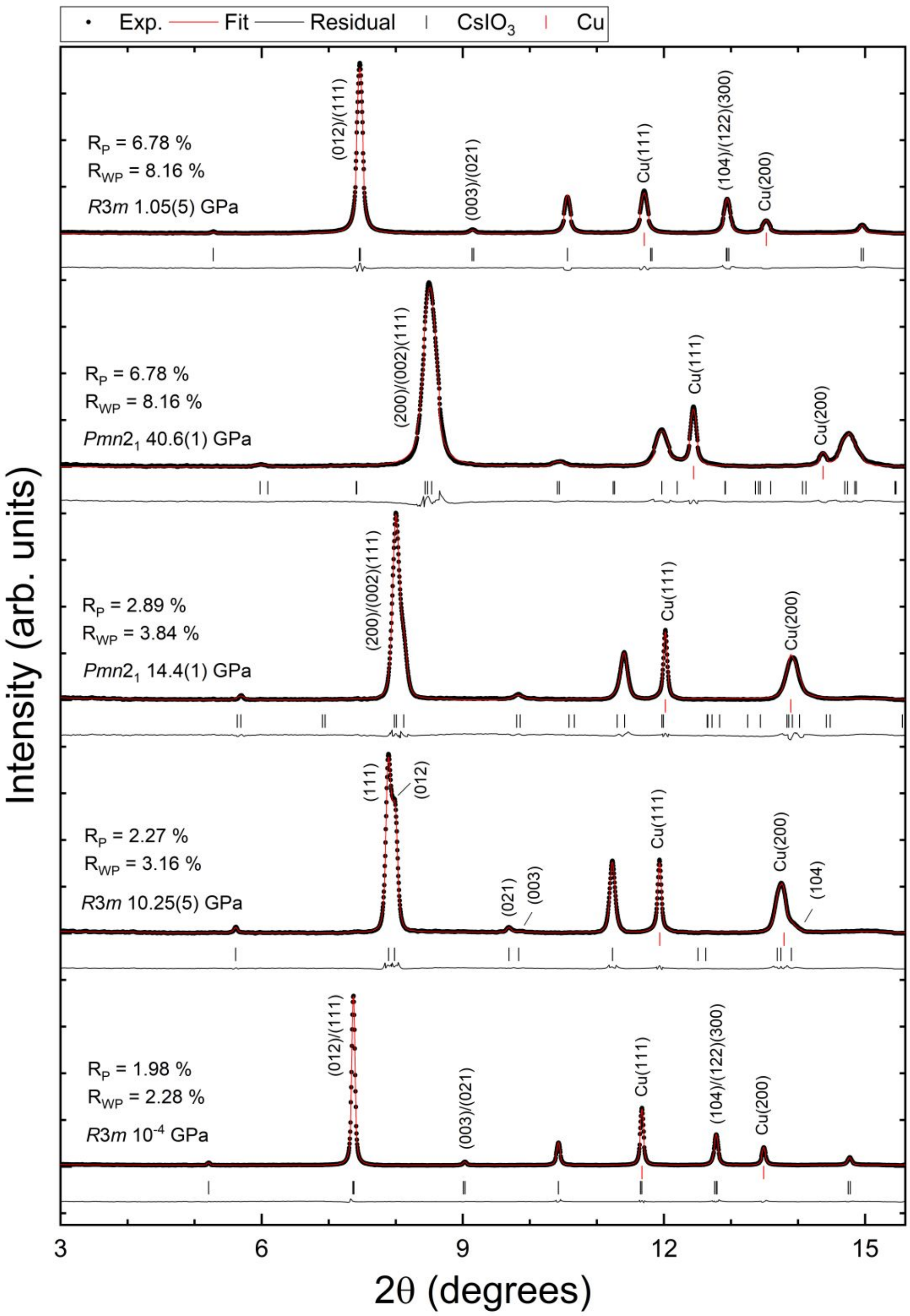

Figure S1. Powder X-ray diffraction patterns measured at selected pressures (λ = 0.4246 Å) in ALBA. Experiments are shown with black symbols. Rietveld refinements with red lines. The residuals with black lines. Black (red) vertical ticks indicate the position of reflections of the sample (Cu). The peaks of Cu used to measure pressure are labeled as well as the peaks from the sample mentioned in the text. R-values of the refinements are also given.

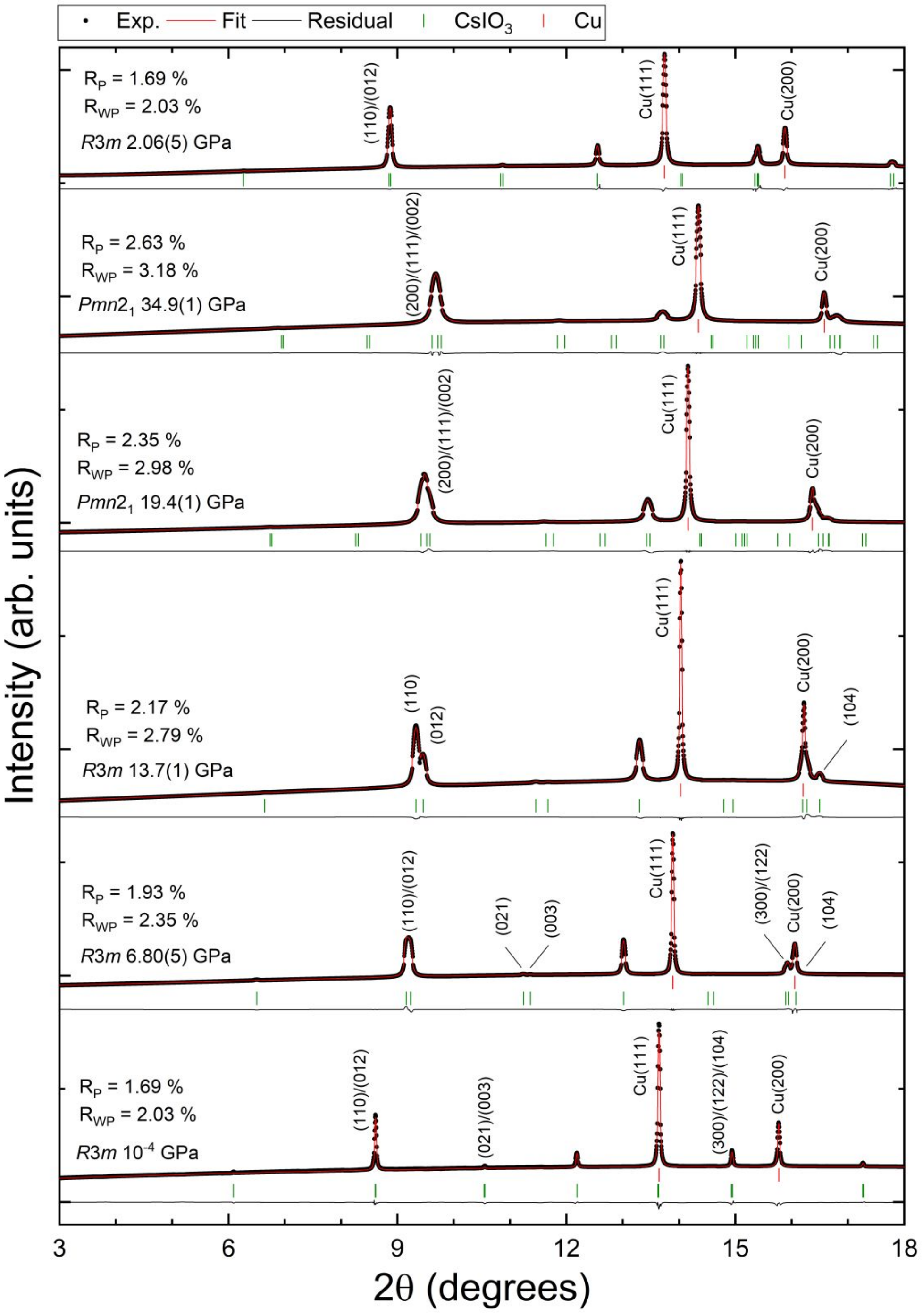

Figure S2. Powder X-ray diffraction patterns measured at selected pressures (λ = 0.4957 Å) in Elettra. Experiments are shown with black symbols. Rietveld refinements with red lines. The residuals with black lines. Black (red) vertical ticks indicate the position of reflections of the sample (Cu). The peaks of Cu used to measure pressure are labeled as well as the peaks from the sample mentioned in the text. R-values of the refinements are also given.

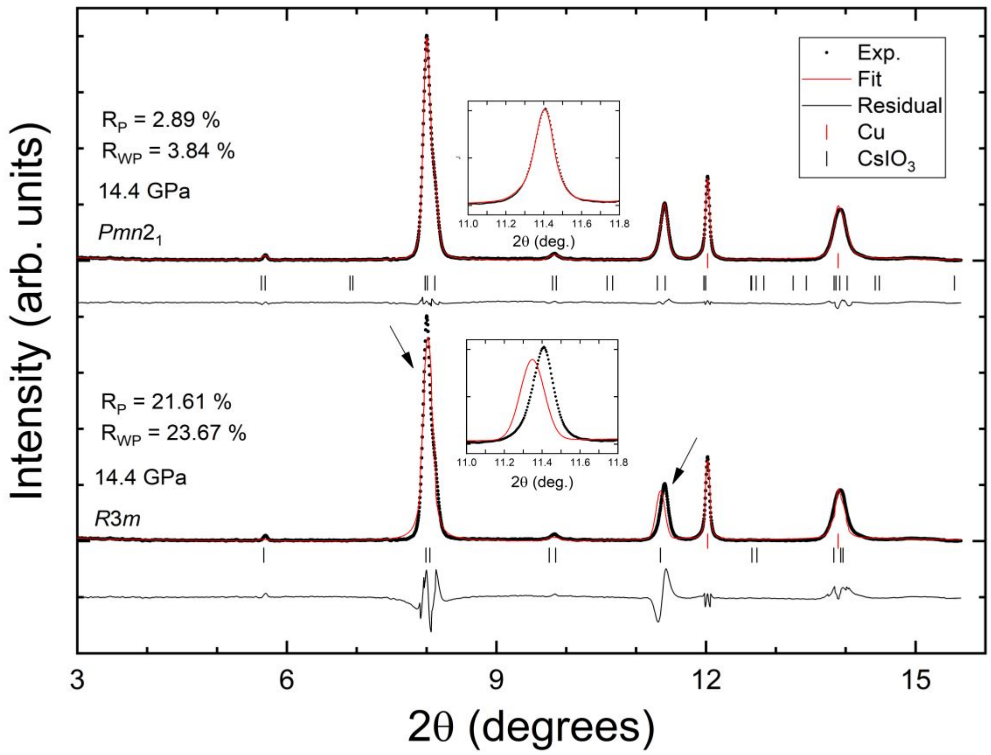

Figure S3. Comparison of Rietveld refinements of the X-ray diffraction pattern (λ = 0.4246 Å) of 14.4(1) GPa assuming the trigonal (space group *R*3*m*) and the orthorhombic (space group *Pmn*2_1) structures as described in the text. Experiments (refinements) are shown with black symbols (red lines). The residuals are plotted as black lines. Black (red) ticks indicate the position of reflections of the sample (Cu). R-values of the refinements are also given. The insets show a zoom of the peak at 11.4° to illustrate the best fit of the orthorhombic structure.

Both rhombohedral and orthorhombic structures are very similar as observed in Figure S3. The crystal structure of the low-pressure phase is related to the high-pressure structure by the transformation matrix $\begin{bmatrix} 2/3 & -2/3 & 1/3 \\ 0 & 0 & 1 \\ 2/3 & 1/3 & 1/3 \end{bmatrix}$. The pressure dependence of the volume for the rhombohedral and orthorhombic phases can be described by a third-order Birch-Murnaghan equation of state (EOS) **[2]**. The experimentally obtained unit-cell volume at zero pressure, bulk modulus and its pressure derivative are V_0 = 305.1(6) Å^3, K_0 = 21.9(9) GPa, K_0' = 7.1(4), which makes $CsIO_3$ one of the most compressible iodates **[3]**, indeed as compressible as $Sr(IO_3)_2HIO_3$ **[4]**, and of the same order as that experimentally estimated in KIO_3 (26.5 GPa) **[5]**. The fitted EOS is shown with a red line in **Fig. 2b**. At the phase transition near 14 GPa, a discontinuity appears in the unit-cell parameters, but not in the volume. In addition, the compressibility becomes more hydrostatic. Noticeably, the EOS of the low-pressure phase describes well also the behavior of the volume of the high-pressure phase. The results from experiments are in good agreement with the results from density functional theory (DFT) calculations (see **Fig. 3** in the main text). The EOS parameters describing the theoretical pressure dependence of the volume for both low-pressure and high-pressure phases are V_0 = 285.3 Å^3, K_0 = 23.2 GPa, and K_0' = 7.3.

Therefore, both the theoretical bulk modulus and its pressure derivative agree with the experimental results within one standard deviation. This theoretical EOS is also valid for the tetragonal *P*4/*nmm* phase above 45 GPa since there is no volume discontinuity between the orthorhombic and tetragonal phases at the phase transition pressure.

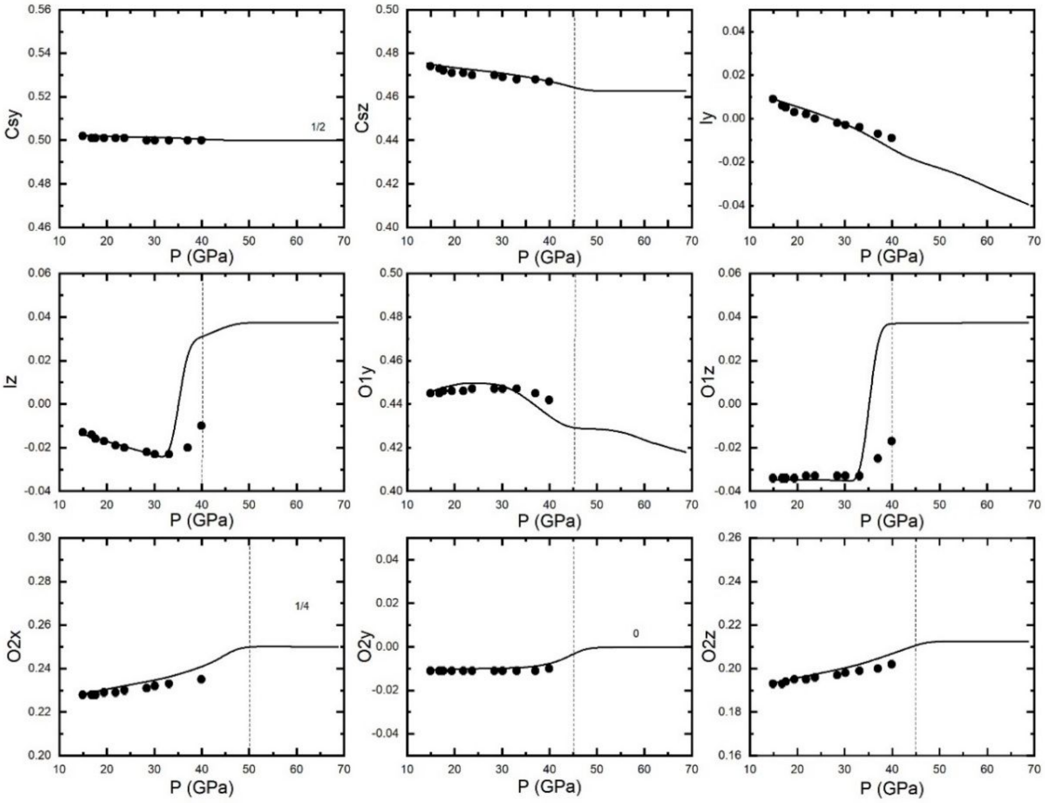

Figure S4. Pressure dependence of experimental (symbols) and theoretical (lines) atomic parameters in the orthorhombic phase of $CsIO_3$. A tendency of some atomic positions towards fixed values, corresponding to high symmetry positions, is observed which leads to a tetragonal phase above 49 GPa.

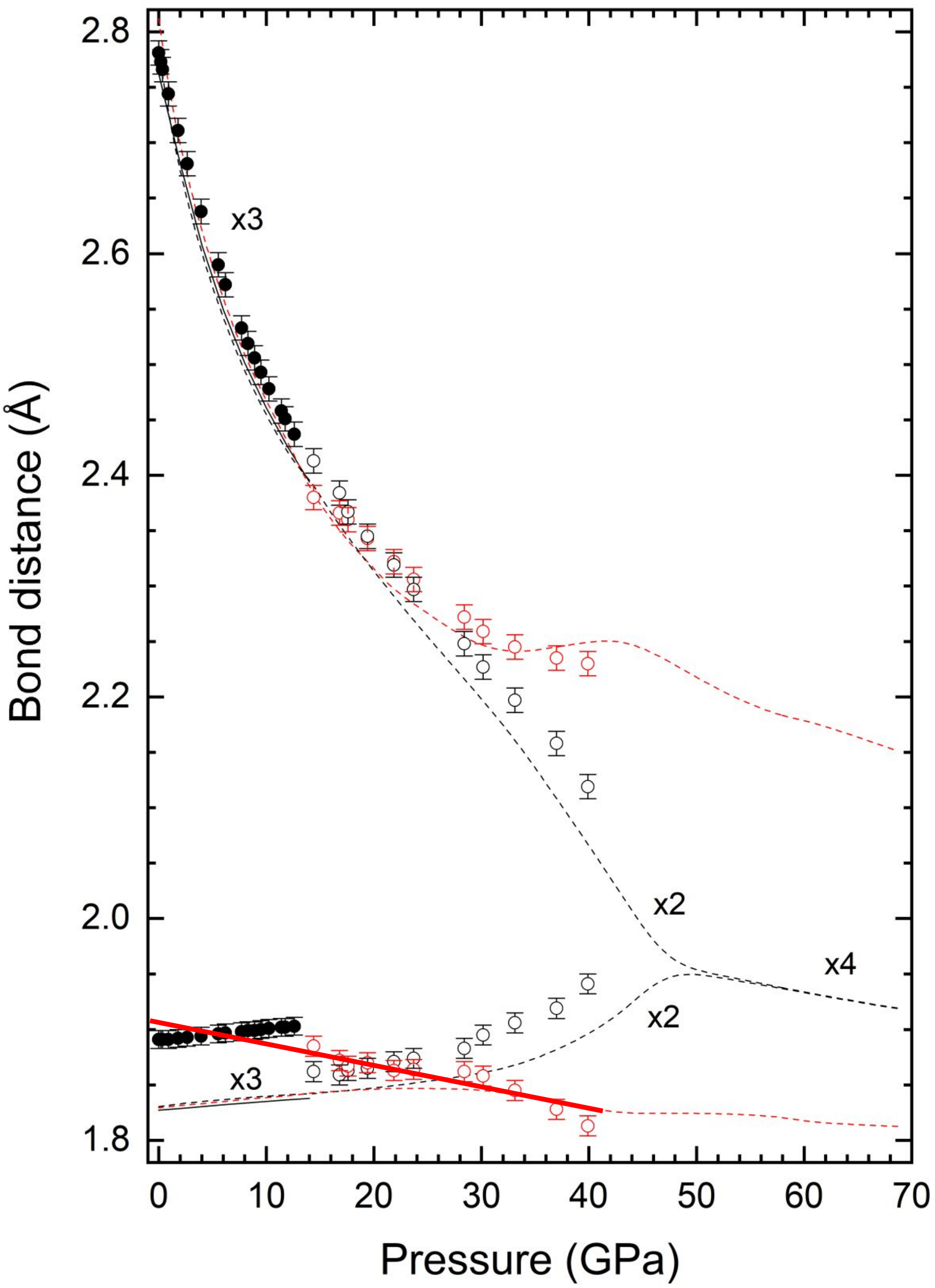

Figure S5. Pressure dependence of the experimental and theoretical I-O bond distances of $CsIO_3$ in the *R*3*m* phase (black solid lines), *Pmn*2$_1$ phase (black and red dashed lines up to 49 GPa), and *P*4/*nmm* phase (black and red dashed lines above 49 GPa). The solid red line represents the tendency of the experimental distances of the delocalized resonant bond (red empty circles) in the *Pmn*2$_1$ phase extrapolated to 0 GPa.

Table S1. Experimental structural information of the rhombohedral crystal structure of $CsIO_3$ at RP.

Space group *R3m*. *a* = 6.606(4) Å, and *c* = 8.102(5) Å.				
Atom	Site	*x*	*y*	*z*
Cs	3a	0	0	0.3122(5)
I	3a	0	0	0.7898(5)
O	9b	0.1401(10)	0.8537(10)	0.6711(10)

Table S2. Experimental structural information of the orthorhombic crystal structure of HP-$CsIO_3$ at 14.4(1) GPa. The CIF file of this structure is deposited at CCDC under Deposition Number 2425042.

Space group $Pmn2_1$. *a* = 6.089(6) Å, *b* = 4.259(4) Å, and *c* = 5.983(6) Å.				
Atom	Site	*x*	*y*	*z*
Cs	2a	0	0.5038(11)	0.4733(11)
I	2a	0	0.0065(11)	0.9873(11)
O1	2a	0	0.4483(20)	0.9659(20)
O2	4b	0.2293(20)	0.9821(20)	0.1937(20)

Table S3. Theoretical structural information of the tetragonal crystal structure of HP-$CsIO_3$ at 52 GPa. Notice that this structure involves a transformation of axes when compared with the orthorhombic structure (a,b,c)→(a,-c,b).

Space group *P4/nmm*. *a* = 5.5027 Å and *c* = 4.0292 Å.				
Atom	Site	*x*	*y*	*z*
Cs	2b	0	0	½
I	2c	0	½	0.02413
O1	2c	0	½	0.57139
O2	4d	¼	¼	0

2. Vibrational properties

A selection of the measured Raman scattering spectra of $CsIO_3$ at different pressures is shown in **Figure S6**. A total of 13 Raman modes has been observed in the range up to 1000 cm^{-1}, in agreement with those previously reported at room pressure **[6]**. According to group theory **[7]**, the *R*3*m* phase (SG 160, Z=1) of $CsIO_3$ should exhibit 15 vibrational modes, comprising 3 acoustic modes (A_1 + E) and 12 optical modes ($3A_1$ + A_2 + 4E), being E modes twofold degenerated. All optical modes are both Raman- and infrared (IR)-active except for the A_2 mode, which is a silent one. This means that both the A_1 and E modes exhibit splitting between transversal optical (TO) and longitudinal optical (LO) modes. Therefore, a total of 22 Raman-active and IR-active modes could be detected in the low-pressure phase of $CsIO_3$ corresponding to 14 different wavenumbers.

The spectrum is characterized by high-wavenumber modes in the region between of 700 and 760 cm^{-1}, which can be assigned to the stretching modes of the IO_3 units as in most iodates **[4,6]**. In addition, Raman modes are present in the medium-wavenumber region between 300 and 400 cm^{-1}, typically corresponding to the bending modes of the IO_3 units as in most iodates. Finally, the Raman modes in the low-wavenumber region below 200 cm^{-1} can be classified as lattice modes and are mainly related to the A cation located among the IO_3 units.

Theoretical calculations (including the TO-LO splitting) have allowed us to tentatively assign the symmetry of the Raman-active modes experimentally observed in the low-pressure phase (see **Table S4**). This assignment allows us to conclude that there are four relatively weak Raman modes that we consider not being first-order modes, but second-order modes of $CsIO_3$ (see green symbols in **Figure 5** in the main text).

According to group theory **[7]**, the orthorhombic *Pmn*2_1 phase (SG No. 31, Z=2) of $CsIO_3$ has 30 vibrational modes, comprising 3 acoustic modes (A_1+ B_1+ B_2) and 27 optical modes ($8A_1$ + $6A_2$ + $5B_1$ + $8B_2$). All optical modes are both Raman- and infrared (IR)-active except for the A_2 modes, which are silent ones. This means that both the A_1, B_1, and B_2 modes exhibit TO-LO splitting. Therefore, a total of 42 Raman- and IR-active modes are expected for the HP phase of $CsIO_3$. However, in our Raman scattering measurements, we have only detected a total of 13 Raman modes between 13.5 and 30.2 GPa.

A tentative assignment is proposed in **Table S5** for the Raman-active modes of the *Pmn*2_1 phase at least between 15 and 25 GPa. Only the lowest-wavenumber mode experimentally observed and having a negative pressure coefficient cannot be attributed to a first-order Raman mode of the orthorhombic phase. We consider that this soft mode is a second-order mode of the high-pressure phase. This is likely a difference mode between a phonon from the region between 300 and 400

cm^{-1} and a phonon from the region between 150 and 240 cm^{-1} since the phonons of the later region have a slightly larger (positive) pressure coefficient than the phonons of the former one.

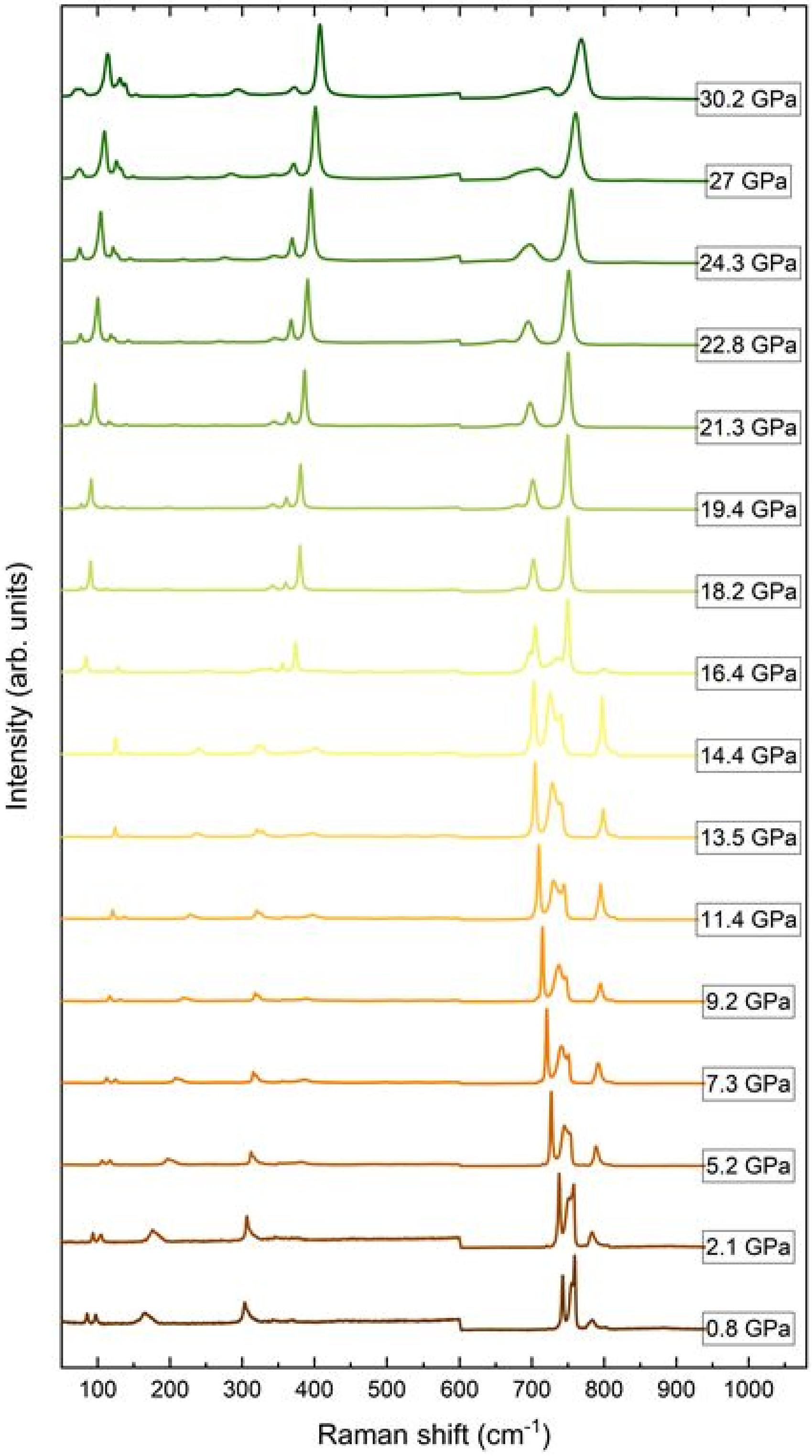

Figure S6. Raman spectra of $CsIO_3$ at selected pressures up to 30.2 GPa. The step near 600 cm^{-1} is an artifact caused by the acquisition program related to overlapping of the two spectral ranges (50-600 cm^{-1} and 600-1000 cm^{-1}) in which the Raman spectra have been measured.

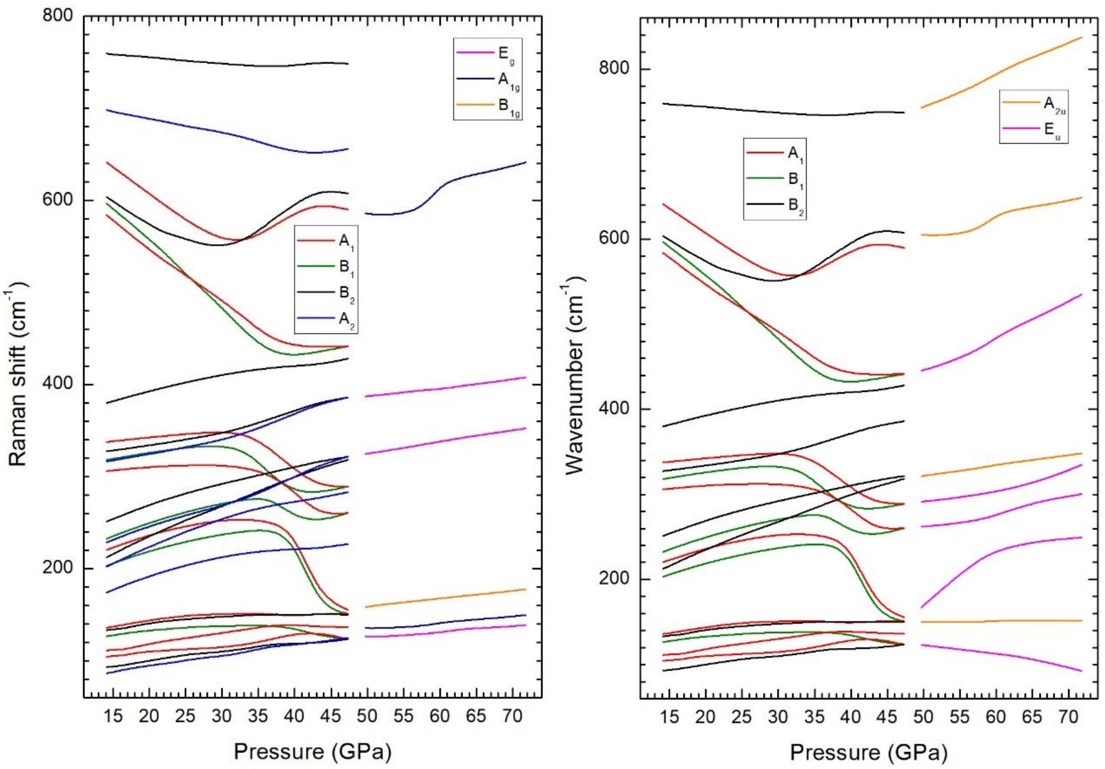

Figure S7. Pressure dependence of theoretical wavenumbers (in cm^{-1}) of the Raman-active (left) and Infrared-active (right) vibrational modes of the *Pmn*2$_1$ phase (up to 47 GPa) and *P*4/*nmm* phase (above 49 GPa) of $CsIO_3$.

Table S4. Theoretical (theo.) and experimental (exp.) Raman-active frequencies at zero pressure (ω_0,in cm^{-1}) and pressure coefficients (b_1, in cm^{-1}/GPa, b_2 in cm^{-1} /GPa^2) in the $R3m$ phase of $CsIO_3$ according to cuadratic fits, $\omega = \omega_0 + b_1 P + b_2 P^2$. Uncertainties are given in parenthesis.

$CsIO_3$ (exp.)				$CsIO_3$ (theo.)		
Mode	$\boldsymbol{\omega_0}$	$\boldsymbol{b_1}$	$\boldsymbol{b_2}$	$\boldsymbol{\omega_0}$	$\boldsymbol{b_1}$	$\boldsymbol{b_2}$
E^1-TO	82.4(6)	5.5(2)	-0.18 (1)	86.6	5.0	-0.16
E^1-LO				87.3	5.4	-0.18
A_1^1	94.1(3)	5.0(2)	-0.10 (1)	96.7	4.8	-0.12
E^2-TO	161.8(8)	7.2(6)	-0.12 (1)	165.2	8.0	-0.20
E^2-LO	162(2)	9.1(2)	-0.26 (4)	172.8	7.7	-0.20
E^3-TO	301.7(7)	2.6(3)	-0.08 (1)	284.7	1.7	-0.04
E^3-LO	307.3(9)	2.0(8)	-0.02 (2)	292.1	1.7	-0.04
A_1^2	339(4)	4.5(1)	-0.18 (9)	339.9	1.2	-0.00
E^4-TO	746.2(2)	4.1(2)	0.07 (1)	697.6	-7.9	0.10
A_1^3	756.8(7)	-2.3(1)	0.01 (1)	724.2	-6.8	0.07
E^4-LO	760.5(5)	-1.4(2)	-0.01 (1)	765.9	-2.0	0.02
Not detected (theo.)	782.4(7)	1.5(6)	-0.03 (1)			
Not detected (theo.)	799(2)	1.2(3)	-0.02 (4)			
Not detected (theo.)	367(1)	3.0(2)	-0.04 (2)			
Not detected (theo.)	726.1(5)	-2.1(2)	0.02 (2)			

Table S5. Theoretical (theo.) and experimental (exp.) Raman-active frequencies at 15 GPa (ω_0,in cm^{-1}) and pressure coefficients (b_1, in cm^{-1}/GPa, b_2 in cm^{-1} /GPa^2) in the $Pmn2_1$ phase of $CsIO_3$ according to cuadratic fits, $\omega = \omega_0 + b_1(P - 15) + b_2(P - 15)^2$. Uncertainties are given in parenthesis.

$CsIO_3$ (exp.)			$CsIO_3$ (theo.)			
$\boldsymbol{\omega_0}$	$\boldsymbol{b_1}$	$\boldsymbol{b_2}$	Mode	$\boldsymbol{\omega_0}$	$\boldsymbol{b_1}$	$\boldsymbol{b_2}$
79(1)	0.0(1)	-0.03(1)	B_2^1-TO	93.1	1.4	-0.02
81(1)	2.8(1)	-0.04(1)	B_2^1-LO	93.2	1.4	-0.01
105(1)	1.8(2)	0.00(1)	A_1^1-TO	105.8	0.6	0.01
105(1)	2.7(3)	-0.02(2)	A_1^1-LO	105.9	0.6	0.01
			A_1^2-TO	110.9	1.5	-0.01
			A_1^2-LO	111.0	1.5	-0.01
126(1)	2.6(1)	-0.05(2)	B_1^1	127.1	1.3	-0.04
			B_2^2-TO	133.6	1.6	-0.03
			B_2^2-LO	134.0	1.5	-0.04
			A_1^3-TO	136.7	1.6	-0.05
			A_1^3-LO	137.3	1.6	-0.04
180(1)	5.1(2)	-0.11(1)	B_1^2	203.9	3.2	-0.07
			B_2^3-TO	214.5	4.7	-0.14
			B_2^3-LO	214.9	4.7	-0.14
			A_1^4-TO	222.1	2.9	0.01
			A_1^4-LO	224.5	2.6	0.02
243(2)	3.2(4)	0.02(2)	B_1^3	235.2	2.8	-0.02
			B_2^4-TO	253.0	3.5	-0.06
			B_2^4-LO	259.7	4.3	-0.10
			A_1^5-TO	307.1	0.6	-0.01
			A_1^5-LO	313.6	0.7	-0.01
			B_1^4	319.8	0.8	0.04
338(2)	1.3(2)	-0.04(1)	B_2^5-TO	324.8	2.9	0.03
			B_2^5-LO	339.3	0.3	-0.11
353(2)	2.7(1)	-0.09(5)	A_1^6-TO	355.4	1.9	-0.05
			A_1^6-LO	374.9	0.5	0.02
371(3)	2.8(2)	-0.02(5)	B_2^6-TO	381.1	2.5	-0.04
			B_2^6-LO	384.2	2.4	-0.04
			A_1^7-TO	580.1	-6.3	0.01
			A_1^7-LO	602.1	-6.8	0.21
			B_1^5	594.2	-7.5	0.00
			B_2^7-LO	604.5	-7.4	0.28
			B_2^7-TO	643.7	-9.3	0.28
713(3)	-4.7(5)	0.34(3)	A_1^8-TO	724.0	-2.7	-0.06
710(3)	-3.8(8)	-0.27(8)	A_1^8-LO	731.2	-1.1	0.01
751(3)	-0.8(1)	0.13(2)	B_2^8-TO	759.1	-0.8	0.01
			B_2^8-LO	763.3	-2.6	0.12

3. Chemical properties

Table S6. Calculated bond distance, Bader charges, and bonding parameters including the number of electrons shared (ES) and the normalized number of electrons transferred (ET) for the short I–O bonds of $CsIO_3$ in the three phases at different pressures to provide insight into the evolution of chemical bonding under compression.

Phase (Pressure, GPa)	bond	distance (Å)	ES	Bader charge	ET
R3m (0)	I–O	1.82716	2.32278	I +2.40<br>O −1.04	0.160
R3m (10)	I–O	1.83511	2.09514	I +2.49<br>O −1.05	0.166
Pmn2$_1$ (20)	I–O	1.84595	1.8876	I +2.62<br>O −1.07	0.175
Pmn2$_1$ (40)	I–O1<br>I–O2	1.82924<br>1.89465	1.74298<br>1.54962	I +2.79<br>O −1.13	0.186
P4/nmm (60)	I–O1<br>I–O2	1.81722<br>1.93372	1.73965<br>1.30733	I +2.79<br>O −1.14	0.186

Table S7. Calculated bond distance, Bader charges, and bonding parameters including the number of electrons shared (ES) and the normalized number of electrons transferred (ET) for the short I–O bonds of $RbIO_3$ in the *R*3*m* and *R*-3*m* phases at 0 and 60 GPa, respectively.

Phase (Pressure, GPa)	bond	distance (Å)	ES	Bader charge	ET
R3m (0)	I–O	1.83551	2.246	I +2.49<br>O −1.07	0.166
R-3m (60)	I–O	1.92155	1.334	I +2.85<br>O −1.10	0.190

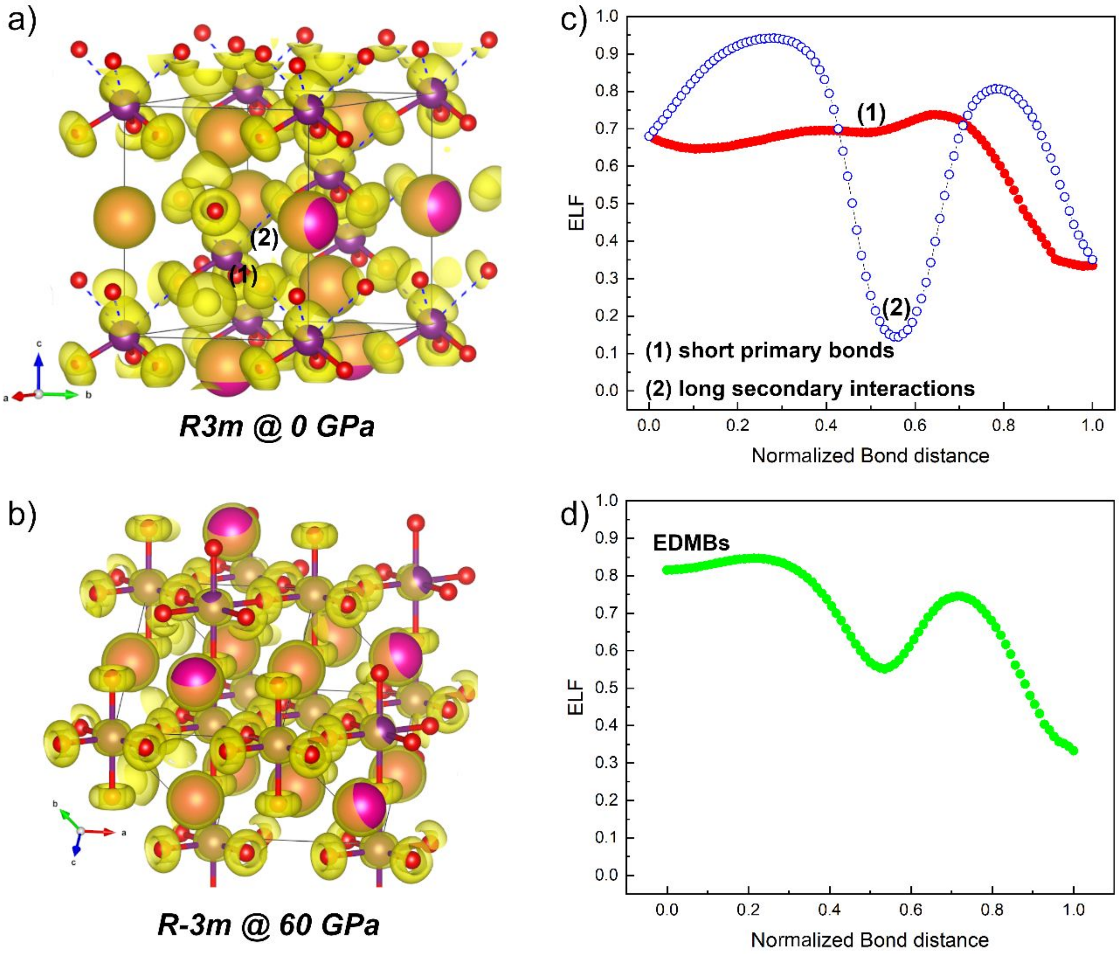

Figure S8. Electron localization function (ELF) isosurfaces and ELF values along the different I–O bonds of $RbIO_3$ for the *R*3*m* phase at 0 GPa (a,c) and the quasi-cubic *R*-3*m* phase at 60 GPa (b,d). In a,b, the yellow isosurfaces correspond to the lone electron pairs (LEP) around I and O atoms, short bonds are depicted with wide bars in red and magenta colors and the long bonds are indicated with dashed blue lines.

4. Bibliography

[1] M. Zhang, C. Hu, T. Abudouwufu, Z. Yang, and S. Pan, *Chem. Mat.* 30, 1136 (2018).

[2] F. Birch, *Phys. Rev.* 71, 809 (1947).

[3] A. Liang, C. Popescu, F.J. Manjón, P. Rodriguez-Hernandez, A. Muñoz, Z. Hebboul, and D. Errandonea, Phys. Rev. B 103, 054102 (2021).

[4] D. Errandonea, H. H. H. Osman, R. Turnbull, D. Diaz-Anichtchenko, A. Liang, J. Sanchez-Martin, C. Popescu, D. Jiang, H. Song, Y. Wang, and F. J. Manjón, Pressure-induced hypercoordination of iodine and dimerization of I_2O_6H in strontium di-iodate hydrogen-iodate ($Sr(IO_3)2HIO_3$). *Materials Today Advances* 22, 100495 (2024).

[5] L. Bayarjargal, L. Wiehl, A. Friedrich, B. Winkler, E.A. Juarez-Arellano, W. Morgenroth, and E. Haussühl, J. Phys.: Condens. Matter 24, 325401 (2012).

[6] E. Salje, *Acta Cryst.* A 32, 233 (1976).

[7] E. Kroumova, M.L. Aroyo, J.M. Perez-Mato, A. Kirov, C. Capillas, S. Ivantchev, H. Wondratschek, *Phase Transitions* 76, 155e170 (2003).
